# Circulating biomarkers and outcome from a randomised phase II trial of sunitinib *vs* everolimus for patients with metastatic renal cell carcinoma

**DOI:** 10.1038/bjc.2016.21

**Published:** 2016-02-23

**Authors:** Martin H Voss, David Chen, Mahtab Marker, A Ari Hakimi, Chung-Han Lee, James J Hsieh, Jennifer J Knox, Maurizio Voi, Robert J Motzer

**Affiliations:** 1 Memorial Sloan Kettering Cancer Center, 353 East 68th Street, New York, NY 10065, USA; 2 Novartis Oncology, One Health Plaza, East Hanover, NJ 07936-1080, USA; 3 Princess Margaret Cancer Center, University of Toronto, 5-210, 610 University Avenue, Toronto, Ontario M5G-2M9, Canada

**Keywords:** renal cell cancer, targeted therapy, biomarker, sunitinib, everolimus

## Abstract

**Background:**

RECORD-3 assessed non-inferiority of progression-free survival (PFS) with everolimus *vs* sunitinib in previously untreated patients with metastatic renal cell carcinoma. Baseline plasma sample collection and randomised design enabled correlation of circulating biomarkers with efficacy.

**Methods:**

Samples were analysed for 121 cancer-related biomarkers. Analyses of biomarkers categorised patients as high or low (*vs* median) to assess association with first-line PFS (PFS1L) for each treatment arm. A composite biomarker score (CBS) incorporated biomarkers potentially predictive of PFS1L with everolimus.

**Results:**

Plasma samples from 442 of the 471 randomised patients were analysed. Biomarkers were associated with PFS1L for everolimus alone (29), sunitinib alone (9) or both (12). Everolimus-specific biomarkers (CSF1, ICAM1, IL-18BP, KIM1, TNFRII) with hazard ratio ⩾1.8 were integrated into a CBS (range 0–5). For CBS low (0–3, *n*=291) *vs* high (4–5, *n*=151), PFS1L differed significantly for everolimus but not for sunitinib. There was no significant difference in PFS1L between everolimus and sunitinib in the high CBS patient cohort.

**Conclusions:**

Baseline levels of multiple soluble biomarkers correlated with benefit from everolimus and/or sunitinib, independent of clinical risk factors. A similar PFS1L was observed for both treatments among patients with high CBS score.

The mainstay of care for patients with metastatic renal cell carcinoma (mRCC) is systemic treatment with molecularly targeted agents. Through exploitation of the universal loss of Von Hippel-Landau (VHL) function with consequent activation of the hypoxia-inducible factor (HIF)/vascular endothelial growth factor (VEGF) axis in conventional RCC, the majority of approved drugs were primarily developed to target tumour angiogenesis (Su *et al*, 2014). Several VEGF receptor (VEGFR)–tyrosine kinase inhibitor (TKI) and mammalian target of rapamycin complex 1 (mTORC1) inhibitors have been approved in the first- (Hudes *et al*, 2007; Motzer *et al*, 2007; Sternberg *et al*, 2010; Motzer *et al*, 2013) and second-line settings (Motzer *et al*, 2008; Rini *et al*, 2011). One example is sunitinib, a multi-TKI with proven progression-free survival (PFS) benefit in patients with mRCC (Motzer *et al*, 2007). Everolimus, approved based on a pivotal study that enrolled patients pretreated with one or two TKIs (Motzer *et al*, 2008), is an allosteric inhibitor of mTORC1. There are no established tissue markers for evidence-based selection of patients who are candidates for treatment with either of these two drug classes. A need for such biomarkers exists in light of the growing number of approved agents available to clinicians.

RECORD-3 (Renal Cell Cancer Treatment With Oral RAD001 Given Daily) was an open-label, randomised, phase II study that compared first-line everolimus followed by second-line sunitinib after documented disease progression and first-line sunitinib followed by everolimus (Motzer *et al*, 2014a). It was designed to test PFS non-inferiority for first-line everolimus compared with first-line sunitinib. The trial did not meet its primary end point. The majority of patients enrolled had clear cell RCC (85%). Although the genomic landscape for this disease should render tumour cells uniformly dependent on VEGF signalling, it is less certain that the trial population was equally homogenous with regard to the extent of underlying activation of mTORC1 signalling. Considering the differences in mechanism of action between these two targeted agents and recognizing that the trial had no molecular selection as entry criteria, we investigated whether first-line PFS (PFS1L) for everolimus might vary within molecularly defined patient subgroups. Specifically, we compared differential PFS1L of everolimus with sunitinib in these patient cohorts by analysing a broad panel of circulating biomarkers using baseline plasma samples collected immediately before initiation of first-line therapy.

## Patients and methods

### Patient population and study design

Details on study design and patient population of RECORD-3 have previously been reported by Motzer *et al* (2014a). The trial enrolled patients with mRCC of clear cell or non-clear cell histology who had not previously received systemic therapy. Everolimus and sunitinib were both administered on standard dosing schedules. Random 1 : 1 assignment was stratified by Memorial Sloan Kettering Cancer Center (MSKCC) risk criteria (Motzer *et al*, 2002). All patients who were included in the RECORD-3 efficacy analysis and from whom the baseline plasma samples had been collected (as per the trial protocol correlative plan) were eligible for the biomarker analysis. RECORD-3 was conducted in accordance with the International Conference on Harmonisation Good Clinical Practice guidelines and approved by the institutional review boards or independent ethics committees of each centre. All patients gave informed consent.

### Sample acquisition and multiplex assay

Approximately 6 ml of peripheral blood for plasma preparation was collected from all patients before the first dose of study drug. A total of 148 candidate soluble proteins associated with angiogenesis, cancer, inflammation, metabolism, tissue remodelling and kidney damage were selected from 20 preconfigured CustomMAP immunoassay panels and measured by a multiplex flow cytometry-based platform by the manufacturer (Multi-Analyte Profile (MAP); Myriad RBM, Austin, TX, USA). A complete list of all 148 candidate biomarkers is included in the Supplementary Appendix (Supplementary Table S1).

### Statistical methods – single-biomarker analysis

Association between PFS1L, first-line study drug treatment and biomarker were investigated separately for each candidate biomarker. Patient samples were dichotomised using the baseline concentration of biomarker. In the initial analysis, samples with biomarker levels at the median or less were categorised as ‘low' for the respective biomarker, whereas samples with levels above the median were classified as ‘high'. Median PFS1L, by first-line treatment and biomarker category, were determined by Kaplan–Meier method. Cox proportional hazards (PH) model compared PFS1L between treatment arms and biomarker category, with stratification by MSKCC risk groups (Motzer *et al*, 2002) and adjustment for baseline covariates (RCC histology, number of metastatic sites, baseline lactase dehydrogenase (LDH) levels). Significance of such associations was tested using the log-rank test, and *P*-values were adjusted for multiple testing over the various biomarkers by applying the Benjamini–Hochberg false discovery rate (FDR) correction method (Benjamini and Hochberg, 1995).

Additional cut points (percentiles) for dichotomisation of baseline biomarker levels were also explored based on a grid search to determine the optimal cutoff for each biomarker. For this, the biomarker high and low groups were redefined as greater than cutoff or less than or equal to cutoff, respectively, using the 20th to 80th percentiles of the baseline level of each biomarker of all samples. The Cox PH models and log-rank tests were computed for each percentile cutoff. Hazard ratios (HRs) between biomarkers (high/low) within each treatment arm and HRs between treatments (everolimus/sunitinib) within each biomarker category were estimated. The log-rank *P*-values were adjusted for multiplicity over the different cutoffs within each biomarker separately via the Benjamini–Hochberg FDR correction method. The optimal cutoff for each biomarker was selected based on the log-rank test FDR-adjusted *P*-value and the estimated HR.

### Composite biomarker score

Biomarkers were deemed potentially predictive of PFS with first-line everolimus if an HR was >1.8 when comparing high (> median) and low (⩽ median) cutoffs (log-rank test, FDR-adjusted *P*-value was <0.05). Five candidate biomarkers met the criteria and were included in a composite biomarker score (CBS) that could be computed for each patient. For each biomarker integrated into the composite score, a value of 1 was assigned if the respective baseline biomarker level fell within the range previously determined to associate with longer PFS1L on single-biomarker analysis (Table 1); a value of 0 was assigned if the respective baseline biomarker level was categorised in the range associated with shorter PFS1L. The sum of the individual values (0 *vs* 1 for each of the five biomarkers) was computed as a composite score for each patient (range 0–5; high=favourable PFS1L). Patients were then dichotomised per composite score as ‘low CBS' (score 0–3) compared with ‘high CBS' (score 4–5). A grid-search algorithm was implemented to determine this cutoff for the CBS. Cox PH model compared PFS1L for high CBS *vs* low CBS, stratified by MSKCC risk groups and adjusted for baseline covariates (RCC histology, number of metastatic sites, baseline LDH); log-rank testing was applied, stratified by MSKCC risk group.

### Interaction with clinical risk grouping

To determine whether the CBS was associated with PFS1L independent of the MSKCC risk stratification, a Cox PH model was fitted, treating the MSKCC groups (Motzer *et al*, 2002) as covariates instead of stratification factors. Attribution of patients to the MSKCC risk group was categorised either as ‘good risk' or ‘intermediate/poor risk' the later categories were combined because of the small number of poor-risk patients. The Cox PH model for PFS1L included terms for treatment, CBS group (0–3 *vs* 4–5), MSKCC group (good *vs* intermediate/poor), all two- and three-way interactions between treatment, MSKCC and CBS group and additional covariates for baseline LDH value, number of metastatic sites and cell histology. HRs comparing the two CBS groups (low, 0–3 *vs* high, 4–5) within each treatment and MSKCC category were estimated from the Cox PH model. Log-rank tests for difference in PFS1L between CBS groups within each first-line treatment and MSKCC risk group were performed. All analyses were performed using SAS, version 9.3 (SAS Institute Inc., Cary, NC, USA).

## Results

### Patient population and candidate biomarkers

**Figure 1:**
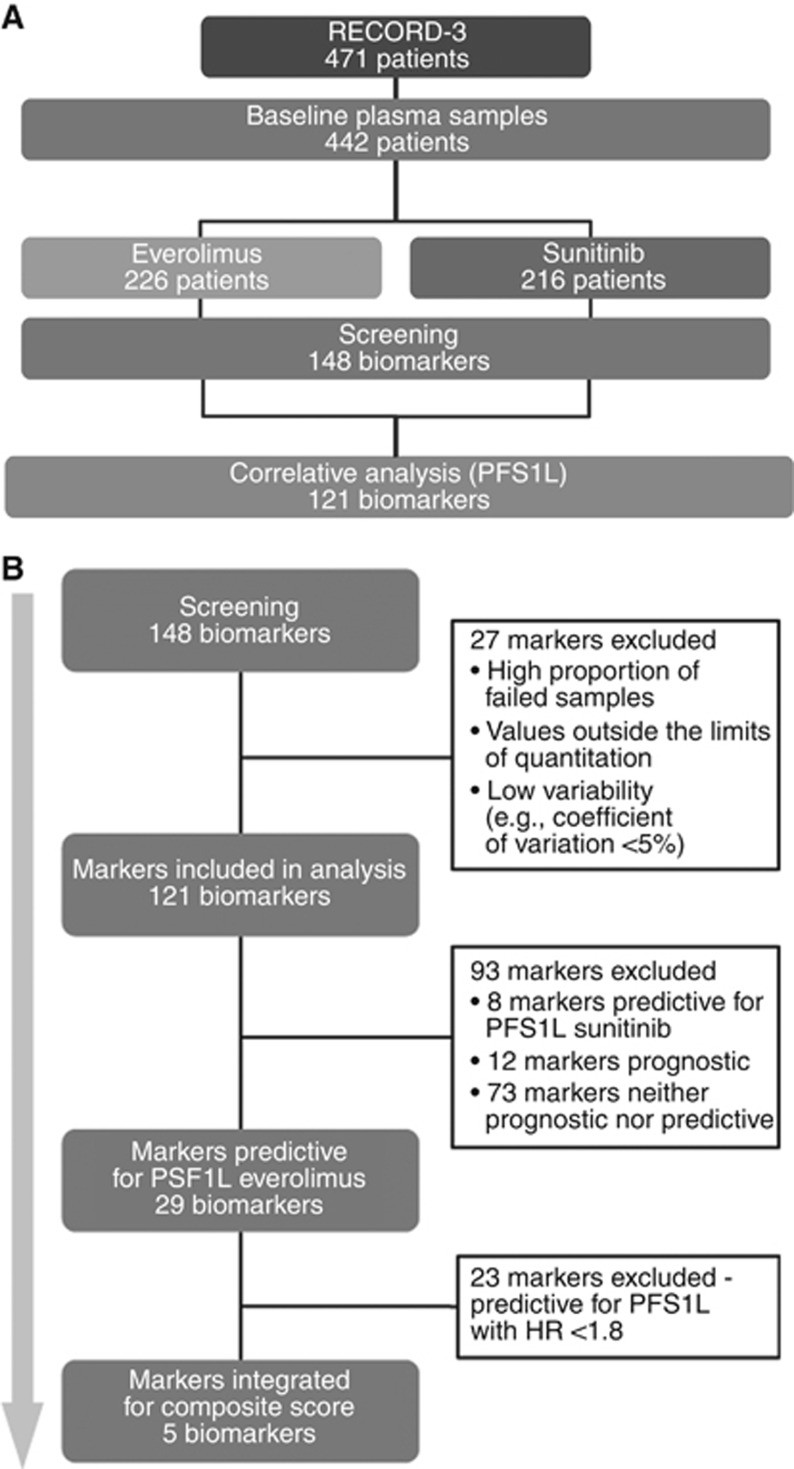
**Study procedure.** (**A**) Patient and biomarker population. (**B**) Development of composite biomarker score. EVE=everolimus; HR=hazard ratio; PFS1L=progression-free survival first line.

Pretreatment plasma samples were available for 442 of the 471 patients enrolled in RECORD-3 (94% of the intent-to-treat (ITT) population), including 226 and 216 patients randomly assigned to receive first-line everolimus or sunitinib, respectively (Figure 1A). Clinical characteristics of the RECORD-3 trial population were previously reported by Motzer *et al* (2014a).

The concentrations of 148 biomarkers were determined (Figure 1B). Of these, 121 biomarkers were included in the subsequent analysis reported here. The remaining 27 biomarkers were excluded from the analyses because of the following: high proportion of failed samples (*n*=3) or high proportion of values (>30%) outside the limits of quantitation or low concentration variability (coefficient of variation <5%) across the biomarker population (*n*=24).

### Biomarker correlations – unsupervised hierarchical clustering

To test a possible correlation between the levels of these biomarkers, values (normalised to have a mean of 0 and an s.d. of 1) were clustered using unsupervised hierarchical clustering with average linkage and Spearman's dissimilarity as a distance measure. Figure 1B shows levels of each of the 121 biomarkers included in the analysis (rows) for individual patients (columns). No apparent clustering was observed (Supplementary Figure S1A). This result remained the same when rare RCC subtypes were removed from the analysis, and unsupervised clustering was limited only to a more homogenous group of 375 patients with conventional clear cell RCC (Supplementary Figure S1B). These results suggest that the biomarker levels are mostly independent and that there is no specific pattern for the RCC subtypes.

### Single-biomarker models – association with PFS1L

An optimal cut point search suggested that dichotomisation into biomarker high compared with biomarker low at or close to the 50th percentile level yielded the most significant segregation of PFS1L benefit (based on FDR adjusted *P*-value) for most of the biomarkers evaluated. Additionally, a 50th percentile cutoff for a biomarker produces more balanced groups in terms of the number of patients in the subgroups. Therefore, the median baseline value was selected as the cut point for each biomarker to define the low and high groups. The biomarkers were then given one of the following five attributions: (i) predictive for everolimus: biomarker level (high *vs* low) only correlates with PFS1L for patients treated with everolimus, not for those treated with sunitinib; (ii) predictive for sunitinib: biomarker level (high *vs* low) only correlates with PFS1L for patients treated with first-line sunitinib, not for those treated with first-line everolimus; (iii) predictive for everolimus and sunitinib both: correlation with PFS1L found for both treatment arms but with opposite effect (high levels favourable for one, unfavourable for the other treatment); (iv) prognostic: biomarker level (high *vs* low) correlates with PFS1L with the same effect (inferior or superior PFS1L) on both treatment arms; and (v) non-prognostic/non-predictive: biomarker level (high *vs* low) has no impact on PFS1L of either treatment arm.

When comparing biomarker high and low populations within each treatment arm, 29 of the 121 candidate biomarkers were deemed predictive of PFS1L with everolimus on the log-rank test (Figure 1B; Table 1; all FDR *P*<0.05). For 23 of these, higher biomarker levels were associated with shorter median PFS1L; for the remaining six biomarkers, higher levels correlated with longer median PFS1L, compared with the median PFS1L of low biomarker subgroup. In contrast, for sunitinib-treated patients, no significant PFS1L difference was found when patients with high and low biomarker levels of these same 29 biomarkers were compared (Table 1). Similarly, nine candidate biomarkers were deemed predictive of PFS1L with sunitinib on a log-rank test (Table 2; all FDR *P*<0.05). For some of these, higher biomarker levels conferred superior outcome within the sunitinib-treated population (e.g., CA153 with PFS1L: HR, 0.551; 95% confidence interval (CI), 0.391–0.775; FDR *P*=0.0094), whereas higher levels of other markers adversely correlated with PFS1L for sunitinib therapy (e.g., ANGPT1 with PFS1L: HR, 1.579; 95% CI, 1.128–2.210; FDR *P=*0.0094; Table 2). Interleukin 18 (IL-18) stood out as the only biomarker with opposite effect on PFS1L for the two treatment arms, thereby meeting criteria to be predictive for everolimus and sunitinib both (Tables 1 and 2). High IL-18 patients on the everolimus arm had shorter PFS1L than low IL-18 patients treated with everolimus (HR, 1.766; 95% CI, 1.298–2.403; FDR *P*=0.0037), whereas high IL-18 patients in the sunitinib arm fared better than low IL-18 patients (HR, 0.6; 95% CI, 0.425–0.847; FDR *P*=0.0498). Therefore, patients with high IL-18 levels had significantly inferior PFS1L with everolimus than with sunitinib (median PFS1L, 5.09 *vs* 12.02 months, respectively; HR, 2.808; 95% CI, 2.010–3.924; FDR *P*<0.0001). Conversely, for patients with low baseline IL-18 level, PFS1L was similar between the treatment arms (median PFS1L, 8.80 *vs* 8.31 months for everolimus and sunitinib, respectively; HR, 0.954; 95% CI, 0.697–1.306; FDR *P*<0.2894).

Eleven candidate biomarkers met the predefined definition of ‘prognostic', whereby comparison of high and low categories suggested statistically significant association with PFS1L for both treatment arms by log-rank test (Table 3; all FDR, *P*<0.05). The remaining 73 candidates were neither prognostic nor predictive, in that the single-biomarker analysis did not show a significant association with PFS1L for either treatment arm. Kaplan–Meier curves for PFS1L, for comparative analysis of the biomarker high and low groups, by treatment arm are included in the Supplementary Appendix for all biomarkers deemed predictive or prognostic (Supplementary Figure S2).

Individual log-rank tests for comparison of PFS1L between treatment arms (sunitinib *vs* everolimus) within the high and low biomarker cohorts were performed for each of the 121 biomarkers. For the majority of subgroups defined by each biomarker, sunitinib was superior in efficacy, as seen in the ITT population. However, baseline levels for 25 of the 29 everolimus-predictive biomarkers and 3 of the 9 sunitinib-predictive biomarkers could molecularly define subgroups of patients for whom the PFS1L was not significantly different between sunitinib and everolimus (log-rank *P*>0.05; Tables 1 and 2).

### CBS – association with PFS1L

To explore whether a multi-biomarker signature would provide a stronger predictive signal, we selected those 5 of the 29 candidate biomarkers with the strongest association with PFS1L (per FDR-adjusted log-rank *P*-value and HR>1.8) in everolimus-treated patients to develop a CBS (Figure 1B). The five biomarkers included were CSF1 (HR, 2.45; 95% CI, 1.77–3.4; FDR *P*<0.0001), ICAM1 (HR, 1.93; 95% CI, 1.41–2.64; FDR *P*=0.0023), IL-18BP (HR, 1.93; CI, 1.41–2.64; FDR *P*=0.0067), KIM1 (HR, 1.83; 95% CI, 1.33–2.51; FDR *P*=0.0009) and TNFRII (HR, 1.89; 95% CI, 1.39–2.57; FDR *P*=0.0037).

For each patient, CBS values were computed from all five biomarkers, and a single CBS value was subsequently determined. A total of 291 (66%) patients of the 442 analysed were categorised as CBS low (score 0–3) and 151 (34%) patients were categorised as CBS high (score 4–5). The optimal cut point search for the CBS also suggested that dichotomisation into CBS high compared with CBS low at the 50th percentile level (CBS 0–3 *vs* 4–5) yielded the most significant segregation of PFS1L benefit (based on FDR-adjusted *P*-value). Table 4 summarises differences in PFS1L per CBS category and treatment arm with HRs (Cox PH model) and the level of significance (log-rank test). Similar to the single-biomarker analyses for the five markers included in the model, significant association between CBS category and PFS1L was found for everolimus-treated patients and not for sunitinib-treated patients.

**Figure 2:**
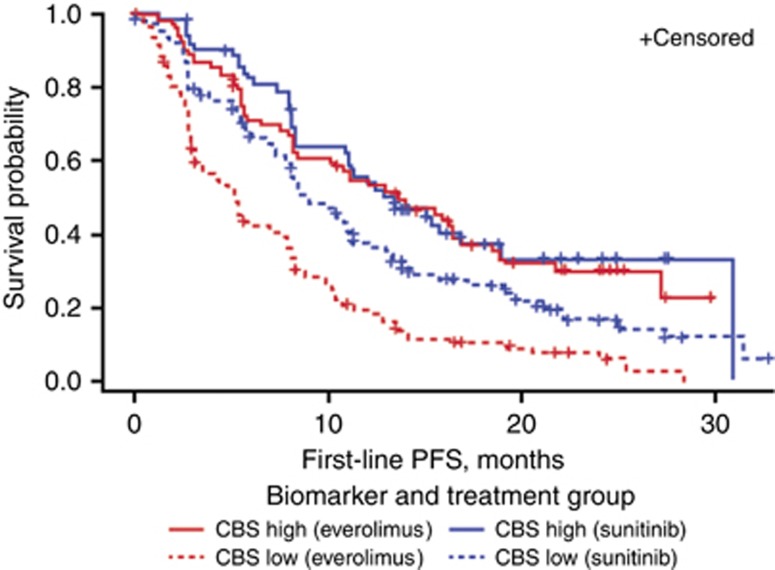
**Kaplan–Meier curves for CBS high compared with low within treatment arms.** CBS=composite biomarker score; PFS=progression-free survival.

Separate analyses were then conducted to compare PFS1L for everolimus and sunitinib within the two CBS categories (CBS high and CBS low). Similar to the result from the ITT population, PFS1L was superior for sunitinib-treated patients within the CBS low group (66% of the study population), in which median PFS1L was 5.13 months for everolimus and 8.77 months for sunitinib (HR, 2.06; 95% CI, 1.57–2.70; *P*<0.0001; Figure 2). However, in the remaining 33% of the patients (CBS high), PFS1L was similar in both treatment arms, with a median PFS of 13.93 months for everolimus and 13.37 months for sunitinib (HR, 1.187; 95% CI, 0.775–1.817; *P*=0.31).

This association of the CBS with PFS1L was further tested by multivariate analysis. A Cox PH model was fit to include the following variables: treatment arm (sunitinib *vs* everolimus); CBS (low, 0–3 *vs* high, 4–5); CBS by treatment arm (CBS high *vs* low; sunitinib *vs* everolimus); RCC histology (clear cell *vs* non-clear cell RCC); and the number of metastatic sites (⩽1 *vs* >1). Findings from the multivariate analysis are summarised in the Supplementary Appendix (Supplementary Table S2). Three factors showed significant association with PFS1L by multivariate testing: number of metastatic sites (*P*<0.0001), treatment arm (*P*<0.0001), and CBS by treatment arm (*P*=0.0321). CBS alone (i.e., without accounting for treatment arm) did not correlate significantly (*P*=0.1399) with PFS1L, providing further support to the notion that the CBS is predictive of everolimus efficacy rather than prognostic for disease progression.

### CBS model – interaction with clinical risk grouping

The MSKCC risk score is a well-established prognostic tool that incorporates clinical and laboratory parameters. Originally developed in a cohort of patients with metastatic RCC treated with cytokine therapy (Motzer *et al*, 2002), it has since been validated for targeted therapies, including sunitinib (Patil *et al*, 2011) and everolimus (Motzer *et al*, 2010). Cox PH modelling with inclusion of CBS category (low=0–3, high=4–5), treatment arm and MSKCC risk group (favourable *vs* intermediate/poor) confirmed association of CBS category with PFS1L on the everolimus treatment arm, both for patients with favourable (HR, 0.43; CI, 0.2–0.75; FDR *P*=0.0094) and those with intermediate/poor MSKCC risk status (HR, 0.40; CI, 0.26–0.61; FDR *P*<0.001). For subjects categorised as CBS high, there was no significant difference in risk for progression with first-line everolimus compared with sunitinib, regardless of MSKCC risk status (MSKCC favourable, *P*=0.4879; MSKCC intermediate/poor, *P*=0.2689). Additional details are included in the Supplementary Appendix (Supplementary Table S3).

## Discussion

Advanced kidney cancer is a model disease for the therapeutic use of molecularly targeted agents. Seven such drugs have been approved by the health authorities in recent years, including five VEFGR inhibitors and two mTOR inhibitors. Despite their well-defined mechanisms of action, no established biomarkers predict clinical efficacy for either of these classes of targeted agents. In the absence of level 1 evidence comparing standard agents in their approved settings, physicians are left to choose between drug classes somewhat arbitrarily, which illustrates the need for biomarkers in this disease. Furthermore, the lack of molecular entry criteria for previously conducted RCC trials for targeted therapeutics raises the question whether the efficacy signal might have been missed in studies deemed negative per their original statistical design. In the current study, we analysed a very broad panel of candidate biomarkers using the baseline plasma samples from 442 patients randomised in a clinical trial of everolimus compared with sunitinib and tested their association with the therapeutic effects of both agents. Although circulating factors have been investigated by others for their association with outcome to VEGFR (Deprimo *et al*, 2007; Tran *et al*, 2012; Zurita *et al*, 2012; Harmon *et al*, 2014; Motzer *et al*, 2014b) and mTOR (Armstrong *et al*, 2012) targeted therapies, this is the first study that compared the two classes of agents. It also investigated the largest number of candidate biomarkers across the biggest cohort of patients thus far.

In a cohort of 68 patients with mRCC treated with first-line sorafenib, previously quantified levels of 52 circulating markers at baseline were evaluated using unsupervised clustering (Zurita *et al*, 2012). Results of the analysis suggested that patients could be dichotomised into two main groups (angiogenic or inflammatory). We were unable to find similar patterns in our larger cohort.

Our study identified that 29 biomarkers exclusively associated with PFS1L everolimus without correlation to therapeutic effects of PFS1L sunitinib. These biomarkers make up a diverse group of molecules with functional significance across various areas of tumour biology, including tissue metabolism (e.g., IGFBP1, leptin), immune response/inflammation (e.g., ICAM1, IL-10, IL-18BP, KIM1, MIP1A), signal transduction (e.g., AXL, HER2ECD), lymphangiogenesis (e.g., SVEGFR3), cell adhesion/extracellular matrix (e.g., FBLN1) and cell death (TRAIL-R3). This heterogeneity likely reflects the span of different cellular processes that mTOR is known to integrate and affect and that these processes are worthy of further investigation as predictive biomarkers for everolimus efficacy (Laplante and Sabatini, 2012). Two principles might account for these potential biomarkers' correlation with everolimus efficacy. Some of the biomarkers might reflect activation of molecular processes that render tumour cells more dependent on mTORC1 signalling and therefore more susceptible to the effects of everolimus. For example, leptin previously has been implicated to activate mTORC1 in obesity (Laplante and Sabatini, 2012) and cancer (Wang *et al*, 2012). In keeping with this, our results suggest that higher circulating levels of leptin were correlated with superior PFS1L (HR, 0.62 for first-line everolimus; Table 1). Alternatively, higher levels of biomarkers could reflect activation of alternate signalling pathways, thus limiting the antitumour effects of inhibition of mTORC1 signalling by everolimus. For instance, higher circulating levels of AXL, a receptor tyrosine kinase and member of the TYRO3, AXL and MERTK (TAM) family, were associated with shorter PFS1L with everolimus (HR, 1.74; Table 1). AXL signals via the PI3K pathway and activates NF*κ*B, MAPK and PLCγ signalling (Hafizi and Dahlback, 2006; Paccez *et al*, 2013).

The five biomarkers that showed the strongest association with therapeutic effect of everolimus were integrated into a composite score with the intent to develop a predictive biomarker for future study. All five biomarkers are functionally involved in inflammation/immune response. We showed significantly better outcomes for everolimus-treated patients with high compared with low CBS, with >2.5-fold longer median PFS1L (HR, 0.43; *P*<0.0001). The distribution of patients between CBS low (66%) and high (34%) shows that a large proportion of patients are in the favourable group, further justifying the need for future validation studies for this signature. For sunitinib-treated patients, the CBS was not significantly associated with outcome (*P*=0.057). These findings were confirmed by multivariate analysis and by subgroup analyses across different MSKCC risk groups. Cox PH calculations comparing outcome with everolimus and sunitinib within CBS biomarker categories revealed a similar PFS1L benefit with both treatments in the CBS high group. This leads us to hypothesise that a subset of patients treated in RECORD-3 (approximately one-third) might have a similar outcome with first-line everolimus, which contrasts with results of the overall analysis of the study performed on the molecularly heterogeneous patients enrolled in the trial. The ability to identify this subgroup of patients would be of high clinical interest in light of the different toxicity profile seen with each agent. Certain co-morbidities (particularly cardiac disease or poorly controlled hypertension) might make treatment with an mTORC1 inhibitor more appealing than the use of a VEGFR-TKI.

In the present analysis, we also identified 9 biomarkers potentially predictive for sunitinib efficacy and 12 molecules that met the definition of prognostic candidate biomarkers for RCC. Although a number of studies have investigated circulating biomarkers for mRCC, most of which used samples from patients treated with a VEGFR-TKI such as pazopanib and sunitinib (Deprimo *et al*, 2007; Pena *et al*, 2010; Tran *et al*, 2012; Zurita *et al*, 2012; Harmon *et al*, 2014), to our knowledge, most biomarkers deemed potentially prognostic in our analysis were not investigated as candidates in these studies. Furthermore, for most of these, the results are limited by their small sample sizes or lack of control arms (i.e., unable to distinguish predictive from prognostic biomarkers). Circulating VEGF-A and VEGF-C and sVEGFR3 have repeatedly been implicated as prognostic biomarkers across several RCC studies (Deprimo *et al*, 2007; Rini *et al*, 2008; Pena *et al*, 2010; Tran *et al*, 2012; Zurita *et al*, 2012). Our findings confirmed circulating VEGF-A as a prognostic biomarker; sVEGFR3 level was only significantly associated with everolimus PFS1L. In a well-controlled analysis, Tran *et al* (2012) studied 21 candidate biomarkers in patients treated with pazopanib after progression during either cytokine- or bevacizumab-containing regimens in a phase II study and validated 7 of these using plasma samples from a placebo-controlled pivotal trial as predictive or prognostic biomarkers. The authors concluded that high IL-6, IL-8, osteopontin, HGF and TIMP1 were poor prognostic biomarkers for PFS. We were able to reproduce these findings in our analysis, including association of shorter PFSL1 with higher baseline levels of IL-8, osteopontin TIMP1 and IL-6 in both arms (Table 3, all *P*-values <0.05). In our analysis, we observed a trend towards a worse outcome in patients with higher baseline levels of HGF; significance was lost after FDR adjustment for multiple testing (everolimus FDR *P*=0.0661; sunitinib FDR *P=*0.0656; Table 2). The nine biomarkers deemed predictive for sunitinib in our analysis were not tested by Tran *et al* (2012) or in other publications noted previously herein. In the subsets of patients identified by three of the nine biomarkers (ANGPTI, CA153, EGFR), the PFS1L superiority of sunitinib compared with everolimus was no longer evident. In the absence of an association with everolimus outcome, one might hypothesise that these biomarkers reflect biological processes that confer relative resistance to sunitinib therapy.

Compared with tumour tissue-based approaches, assessment of biomarkers from peripheral blood is attractive for several reasons: sample acquisition is minimally invasive to patients, and the model proposed in this study only requires a single blood draw. In contrast with tumour tissue, blood samples are easily obtained immediately before treatment initiation, therefore providing information on tumour and host biology in real time. Concerns over misinterpreting findings because of sampling errors in the background of tumour heterogeneity (Gerlinger *et al*, 2014) are of lesser concern with this approach because the molecular phenotype assessed from peripheral blood should be representative of systemically disseminated clones. An obvious shortcoming of using circulating biomarkers to study these cancers is that they only indirectly reflect disease biology, making it more challenging to understand the underlying molecular processes in tumour cells and their microenvironment. In that regard, identification of novel targets for therapeutic intervention would ultimately prove more difficult.

Herein we provide the first report to correlate outcome with mTORC1 inhibitor therapy and soluble biomarkers and the first to compare the prognostic/predictive significance of such markers across two classes of molecularly targeted agents. The strength of this analysis lies in its large samples size, the randomised trial design, the hypothesis-free approach evaluating a large number of candidate biomarkers across various areas of both tumour and host biology and the randomised trial design, which provides opportunity to compare the correlation of biomarker levels across the two standard classes of agents used in this disease. By investigating the largest panel of biomarkers tested in an RCC cohort thus far, this effort yielded more candidates for predictive and prognostic biomarkers than any prior reports.

The current study has several limitations. Although the broad biomarker panel used here increased sensitivity, it concurrently increased the risk for false-positive signals, despite multiplicity adjustments. Independent validation and a prospective study will be essential to confirm our findings and to establish the clinical usefulness of any of the single biomarkers or the CBS. Not all biomarkers included in the original panel could be integrated into our analysis because of assay data quality. Finally, given that RECORD-3 randomly assigned patients to two therapeutic interventions, one must consider the possibility (albeit rather unlikely) that biomarkers deemed prognostic per our analysis might be predictive for everolimus and sunitinib. The only way to test this would be to validate our findings with samples from a placebo-controlled study.

In conclusion, this study, via analysis of plasma samples obtained before initiation of systemic targeted therapy, identified a number of circulating biomarkers that could provide prognostic and predictive information for treatment using everolimus and sunitinib in mRCC. These should be studied prospectively and might ultimately prove effective in interpreting efficacy data or stratifying patients who might participate in future clinical trials. The biological mechanisms behind such correlation are largely unclear and warrant future study to further our understanding of treatment resistance, both primary and acquired. A five-biomarker CBS was predictive of PFS with first-line everolimus with both univariate and multivariate analyses, and it provides predictive information across all the MSKCC risk groups. Although RECORD-3 confirmed VEGFR-TKI therapy as a standard for first-line treatment for unselected treatment-naive patients, this biomarker signature, if validated prospectively, might justify use of everolimus in the same setting for patients at high risk for TKI-related toxicity. Similarly, additional studies might prove the CBS useful in directing treatment choice in the second-line setting.

## Figures and Tables

**Table 1:** Single-biomarker analysis of candidate cytokines predictive of PFS with EVE: Kaplan–Meier analyses for PFS by treatment arm (everolimus *vs* sunitnib) and biomarker category (low *vs* high)

	**PFS1L comparison high** ***vs*** **low marker level within each treatment arm**		**PFS1L comparison EVE** ***vs*** **SUN treatment arm by high** ***vs*** **low marker level**	
**Marker**	**EVE: HR (95% CI) (FDR** ***P***)	**SUN: HR (95% CI) (FDR** ***P***)	**Low: HR (95% CI) (FDR** ***P***)	**High: HR (95% CI) (FDR** ***P***)
SixCKine	1.407 (1.035–1.912) (0.0280)	0.747 (0.536–1.040) (0.1471)	1.149 (0.834–1.583) (0.4314)	2.163 (1.569–2.983) (0.0009)
ACE	0.625 (0.459–0.852) (0.0487)	0.719 (0.513–1.009) (0.0732)	1.691 (1.245–2.297) (0.0127)	1.470 (1.051–2.056) (0.0627)
AXL	1.735 (1.271–2.369) (0.0108)	1.073 (0.76–1.498) (0.4172)	1.339 (0.991–1.808) (0.1188)	2.16 (1.535–3.053) (0.0022)
CA9	1.587 (1.166–2.159) (0.0275)	0.921 (0.660–1.286) (0.4681)	1.256 (0.920–1.714) (0.1872)	2.162 (1.554–3.007) (0.0026)
CARCIEA1	1.662 (1.222–2.260) (0.0124)	0.939 (0.667–1.323) (0.445)5	1.214 (0.892–1.652) (0.1934)	2.149 (1.53–3.012) (0.0032)
CARCIEA6	1.642 (1.207–2.233) (0.0226)	1.491 (1.067–2.083) (0.0610)	1.535 (1.094–2.153) (0.0365)	1.690 (1.248–2.289) (0.0126)
CCL20	1.701 (1.248–2.319) (0.0029)	1.190 (0.850–1.668) (0.2471)	1.344 (0.962–1.878) (0.1806)	1.921 (1.408–2.620) (0.0015)
CLEC3B	0.593 (0.430–0.816) (0.0075)	0.814 (0.581–1.140) (0.1349)	1.793 (1.333–2.411) (0.0066)	1.306 (0.914–1.866) (0.1430)
CSF1	2.449 (1.769–3.391) (<0.000)	1.417 (1.004–1.999) (0.0616)	1.276 (0.904–1.800) (0.1878)	2.205 (1.627–2.989) (0.0008)
CTSB	1.443 (1.065–1.955) (0.0347)	1.246 (0.891–1.743) (0.0861)	1.513 (1.093–2.096) (0.0268)	1.753 (1.275–2.409) (0.0166)
EZR	1.608 (1.178–2.195) (0.0094)	1.067 (0.750–1.517) (0.2178)	1.300 (0.941–1.797) (0.1466)	1.959 (1.423–2.697) (0.0028)
FBLN1	0.615 (0.452–0.836) (0.0175)	0.873 (0.626–1.217) (0.2729)	1.871 (1.366–2.563) (0.0066)	1.318 (0.951–1.827) (0.1213)
GPI	1.511 (1.103–2.071) (0.0180)	1.056 (0.748–1.492) (0.2197)	1.322 (0.957–1.825) (0.1045)	1.891 (1.374–2.602) (0.0046)
GSN	0.581 (0.424–0.795) (0.0094)	0.838 (0.595–1.179) (0.1556)	1.874 (1.368–2.56) (0.0066)	1.299 (0.934–1.80) (0.1444)
HER2ECD	0.661 (0.485–0.900) (0.0226)	0.667 (0.476–0.933) (0.0611)	1.584 (1.161–2.162) (0.0156)	1.570 (1.129–2.184) (0.0499)
ICAM1	1.928 (1.407–2.641) (0.0023)	1.116 (0.796–1.56) (0.3496)	1.250 (0.900–1.735) (0.2614)	2.159 (1.579–2.952) (0.0012)
IGFBP1	1.517 (1.114–2.066) (0.0067)	1.180 (0.844–1.650) (0.1150)	1.387 (1.003–1.918) (0.0816)	1.783 (1.298–2.449) (0.0077)
IITCAC	1.563 (1.148–2.128) (0.0226)	1.193 (0.852–1.669) (0.1123)	1.374 (0.984–1.918) (0.0715)	1.801 (1.324–2.450) (0.0072)
IL-10	1.690 (1.235–2.31) (0.0127)	0.972 (0.690–1.370) (0.2560)	1.211 (0.872–1.681) (0.1288)	2.105 (1.533–2.890) (0.0028)
IL-18	1.766 (1.298–2.403) (0.0037)	0.600 (0.425–0.847) (0.0498)	0.954 (0.697–1.30) (0.2894)	2.808 (2.010–3.924) (<0.0001)
IL-18BP	1.927 (1.406–2.642) (0.0067)	1.058 (0.759–1.475) (0.3976)	1.215 (0.879–1.678) (0.2521)	2.213 (1.609–3.044) (0.0012)
KIM1	1.828 (1.329–2.514) (0.0009)	1.410 (1.002–1.985) (0.0520)	1.401 (1.003–1.956) (0.1404)	1.815 (1.335–2.470) (0.0041)
LEPTIN	0.616 (0.452–0.840) (0.0175)	0.863 (0.618–1.204) (0.2729)	1.873 (1.371–2.559) (0.0061)	1.337 (0.962–1.858) (0.1288)
MIP1A	1.664 (1.226–2.260) (0.0127)	1.099 (0.786–1.536) (0.3976)	1.318 (0.952–1.826) (0.1275)	1.997 (1.455–2.741) (0.0026)
NRP1	1.723 (1.252–2.372) (0.0067)	1.294 (0.915–1.828) (0.0732)	1.429 (1.015–2.013) (0.0616)	1.904 (1.400–2.588) (0.0026)
PRL	1.542 (1.131–2.101) (0.0450)	0.898 (0.645–1.251) (0.4455)	1.210 (0.875–1.671) (0.3279)	2.076 (1.507–2.861) (0.0026)
SVEGFR3	1.570 (1.150–2.144) (0.0127)	1.047 (0.747–1.468) (0.2702)	1.279 (0.924–1.770) (0.1608)	1.917 (1.392–2.640) (0.0058)
TNFRII	1.888 (1.388–2.569) (0.0037)	1.014 (0.725–1.417) (0.3017)	1.215 (0.884–1.669) (0.1872)	2.262 (1.639–3.123) (0.0011)
TRAIL3	1.575 (1.163–2.134) (0.0383)	1.273 (0.914–1.773) (0.1360)	1.429 (1.037–1.971) (0.0550)	1.769 (1.285–2.434) (0.0156)

Abbreviations: CI=confidence interval; EVE=everolimus; FDR=false discovery rate; HR=hazard ratio; PFS=progression-free survival; PFS1L=progression-free survival first line; SUN=sunitinib.

A low CBS score is 0–3 and a high CBS score is 4–5.

**Table 2:** Single-biomarker analysis of candidate cytokines predictive of PFS with SUN: Kaplan–Meier analyses for PFS by treatment arm (everolimus *vs* sunitnib) and biomarker category (low *vs* high)

	**PFS1L comparison high** ***vs*** **low marker level within each treatment arm**		**PFS1L comparison EVE** ***vs*** **SUN treatment arm by high** ***vs*** **low marker level**	
**Marker**	**EVE: HR (95% CI) (FDR** ***P***)	**SUN: HR (95% CI) (FDR** ***P***)	**Low: HR (95% CI) (FDR** ***P***)	**High: HR (95% CI) (FDR** ***P***)
ANGPT1	1.278 (0.943–1.733) (0.2178)	1.579 (1.128–2.210) (0.0094)	1.765 (1.265–2.464) (0.0077)	1.430 (1.049–1.949) (0.1229)
CA153	1.211 (0.889–1.649) (0.4139)	0.551 (0.391–0.775) (0.0094)	1.058 (0.775–1.444) (0.4492)	2.325 (1.671–3.235) (0.0009)
CCL5	1.440 (1.060–1.957) (0.1070)	1.432 (1.016–2.020) (0.0234)	1.555 (1.116–2.165) (0.0311)	1.563 (1.145–2.134) (0.0399)
EGFR	0.676 (0.495–0.924) (0.1941)	0.513 (0.364–0.724) (0.0017)	1.414 (1.042–1.919) (0.1236)	1.862 (1.321–2.626) (0.0066)
FERRITIN	1.388 (1.018–1.892) (0.0590)	1.524 (1.090–2.130) (0.0226)	1.663 (1.189–2.326) (0.0249)	1.514 (1.117–2.052) (0.0153)
IL-18	1.766 (1.298–2.403) (0.0037)	0.600 (0.425–0.847) (0.0498)	0.954 (0.697–1.306) (0.2894)	2.808 (2.010–3.924) (<0.000)
KLK5	0.720 (0.529–0.979) (0.1510)	0.666 (0.474–0.935) (0.0224)	1.532 (1.121–2.094) (0.0487)	1.657 (1.194–2.300) (0.0096)
SLPI	1.429 (1.050–1.945) (0.0972)	1.426 (1.017–1.998) (0.0234)	1.563 (1.123–2.176) (0.0200)	1.567 (1.149–2.137) (0.0324)
TNC	1.371 (1.001–1.876) (0.0661)	1.552 (1.108–2.175) (0.0175)	1.663 (1.183–2.338) (0.0324)	1.468 (1.083–1.990) (0.0337)

Abbreviations: CI=confidence interval; EVE=everolimus; FDR=false discovery rate; HR=hazard ratio; PFS=progression-free survival; PFS1L=progression-free survival first line; SUN=sunitinib.

A low CBS score is 0–3 and a high CBS score is 4–5.

**Table 3:** Single-biomarker analysis of candidate prognostic cytokines: Kaplan–Meier analyses for PFS by treatment arm (everolimus *vs* sunitnib) and biomarker category (low *vs* high)

	**PFS1L comparison high** ***vs*** **low marker level within each treatment arm**		**PFS1L comparison EVE** ***vs*** **SUN treatment arm by high** ***vs*** **low marker level**	
**Marker**	**EVE: HR (95% CI) (FDR** ***P***)	**SUN: HR (95% CI) (FDR** ***P***)	**Low: HR (95% CI) (FDR** ***P***)	**High: HR (95% CI) (FDR** ***P***)
CALBIDN	2.189 (1.567–3.057) (<0.0001)	1.479 (1.048–2.086) (0.0121)	1.301 (0.931–1.819) (0.1674)	1.926 (1.415–2.623) (0.0015)
CCL23	1.673 (1.217–2.300) (0.0226)	1.766 (1.256–2.483) (0.0094)	1.626 (1.157–2.283) (0.0365)	1.540 (1.137–2.087) (0.0177)
CTSD	1.473 (1.082–2.005) (0.0226)	1.422 (1.012–1.999) (0.0127)	1.540 (1.099–2.158) (0.0387)	1.594 (1.176–2.161) (0.0238)
CYSTANB	1.705 (1.249–2.327) (0.0094)	1.521 (1.091–2.120) (0.0414)	1.508 (1.082–2.101) (0.0536)	1.690 (1.239–2.307) (0.0177)
IL-6	1.936 (1.405–2.666) (NA^a^)	1.931 (1.362–2.736) (NA^a^)	1.582 (1.122–2.23) (NA^a^)	1.586 (1.173–2.145) (NA^a^)
IL-8	2.491 (1.798–3.451) (<0.0001)	1.724 (1.233–2.408) (0.0014)	1.260 (0.895–1.774) (0.1833)	1.821 (1.343–2.469) (0.0084)
OSTEOPTN	2.071 (1.485–2.889) (<0.0001)	1.581 (1.116–2.239) (0.0059)	1.401 (0.992–1.977) (0.1000)	1.835 (1.358–2.480) (0.0026)
SPINK1	1.781 (1.303–2.433) (0.0067)	1.673 (1.195–2.342) (0.0103)	1.599 (1.138–2.246) (0.0249)	1.702 (1.257–2.304) (0.0130)
TIMP1	2.404 (1.752–3.298) (<0.0001)	1.511 (1.067–2.138) (0.0049)	1.241 (0.882–1.746) (0.2077)	1.975 (1.449–2.691) (0.0052)
VCAM1	1.644 (1.205–2.243) (0.0303)	1.577 (1.126–2.209) (0.0162)	1.605 (1.143–2.254) (0.0174)	1.673 (1.231–2.275) (0.0139)
VEGF	2.160 (1.566–2.979) (0.0014)	1.672 (1.191–2.346) (0.0053)	1.378 (0.979–1.941) (0.0616)	1.781 (1.314–2.414) (0.0128)
WFDC2	1.882 (1.369–2.588) (0.0017)	1.427 (1.018–2.001) (0.0368)	1.422 (1.016–1.989) (0.0944)	1.875 (1.380–2.547) (0.0028)

Abbreviations: CI=confidence interval; EVE=everolimus; FDR=false discovery rate; HR=hazard ratio; NA=not applicable; PFS=progression-free survival; PFS1L=progression-free survival first line; SUN=sunitinib.

A low CBS score is 0–3 and a high CBS score is 4–5.

a IL-6 had ∼40% of the values below the lower limit of quantitation and was not included in the screening set for further analysis.

**Table 4:** Performance of the CBS: Kaplan–Meier analyses for PFS1L by CBS category and treatment arms

**Comparison**	**Subgroup**	**Median PFS1L, months**	**HR**	**95% CI for HR**	**FDR** ***P*****-value**
CBS high *vs* low^a^	EVE	13.93 *vs* 5.13	0.432	0.307–0.606	<0.0001
CBS high *vs* low^a^	SUN	13.17 *vs* 8.77	0.748	0.509–1.100	0.0570
EVE *vs* SUN	CBS high	13.93 *vs* 13.17	1.187	0.775–1.817	0.3080
EVE *vs* SUN	CBS low	5.13 *vs* 8.77	2.059	1.570–2.699	<0.0001

Abbreviations: CBS=composite biomarker score; CI=confidence interval; EVE=everolimus; FDR=false discovery rate; HR=hazard ratio; PFS1L=progression-free survival first line; SUN=sunitinib.

a A low CBS score is 0–3 and a high CBS score is 4–5.
