# Supplementary material for: Circulating biomarkers and outcome from a randomised phase II trial of sunitinib vs everolimus for patients with metastatic renal cell carcinoma
Source: Br J Cancer. 2016 Feb 23;114(6):642–9. doi: 10.1038/bjc.2016.21 (PMC4800293; doi:10.1038/bjc.2016.21)
Supplement: Supplementary Figure Legends [file bjc201621x3.pdf]

Supplemental Appendix

FIGURE LEGEND

Figure 1. Unsupervised hierarchical clustering of circulating biomarkers A) among patients with mRCC of any histology and B) within the subgroup of patients with clear cell mRCC. mRCC, metastatic renal cell carcinoma.

SupFig1

A

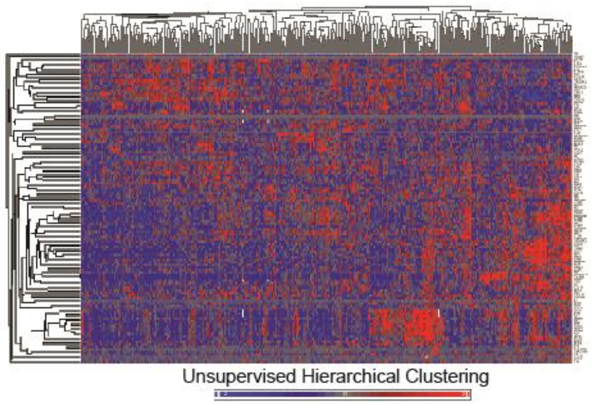

B

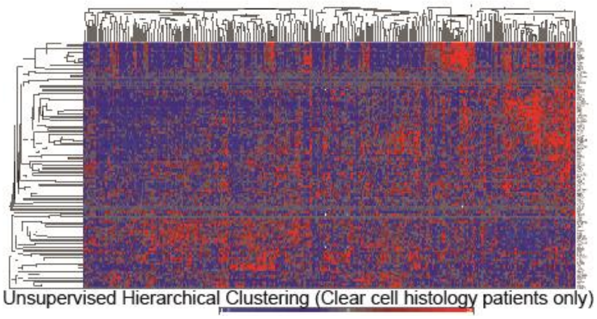

FIGURE LEGEND

Figure 2. Kaplan-Meier curves of PFS1L for individual marker analyses within treatment arms comparing high (>50th percentile) vs. low (≤50th percentile) for predictive and prognostic markers (50 separate curves)

Everolimus Predictive biomarkers

SupFIG2_01 SixCKINE

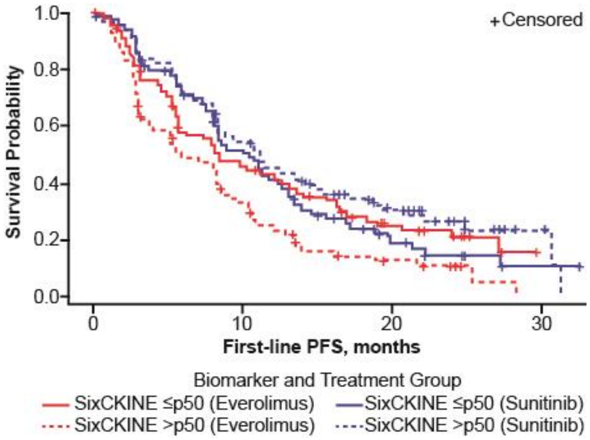

SupFIG2_02_ACE

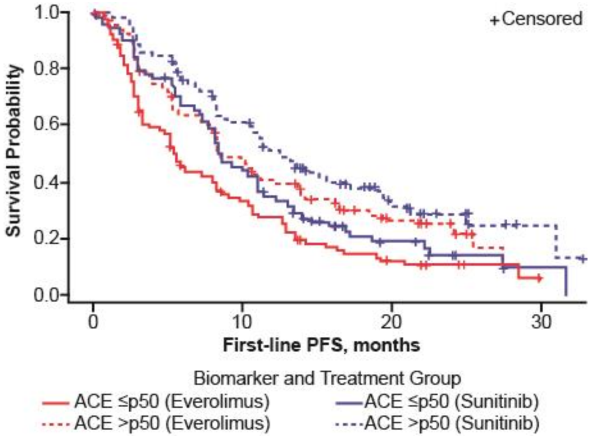

SupFIG2_03 AXL

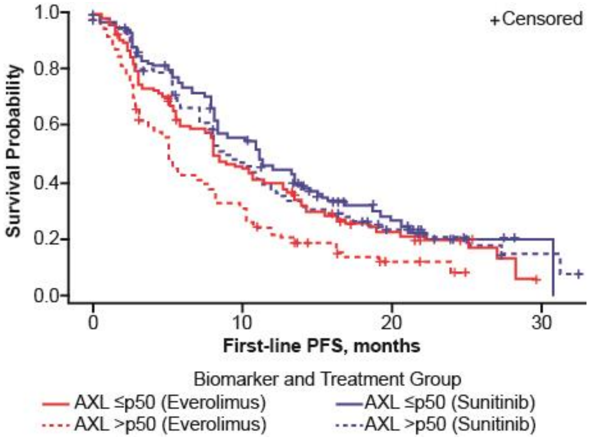

SupFIG2_04 CA9

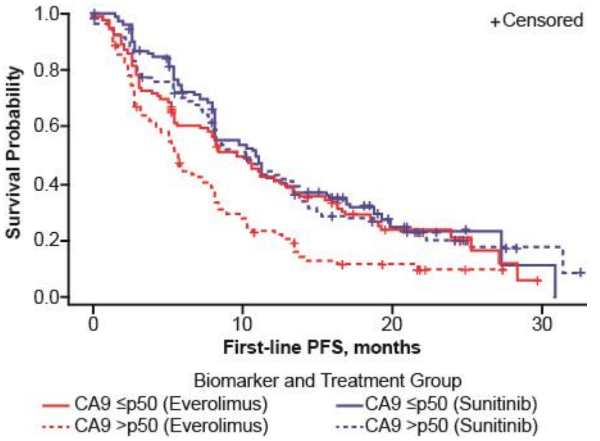

SupFIG2_05 CARCIEA1

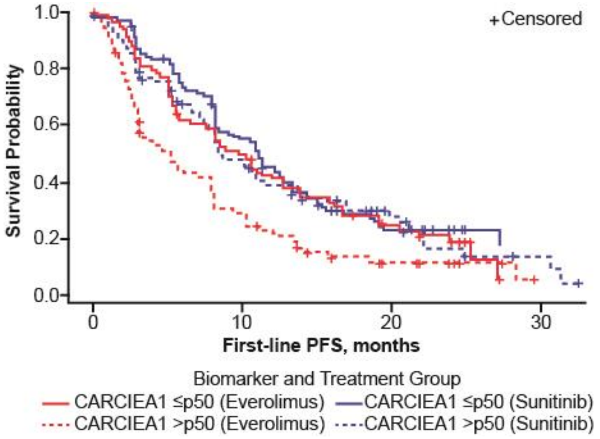

SupFIG2_06_CARCIEA6

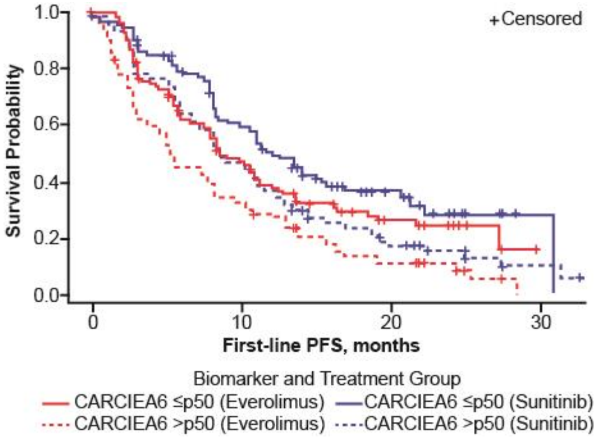

SupFIG2_07_CCL20

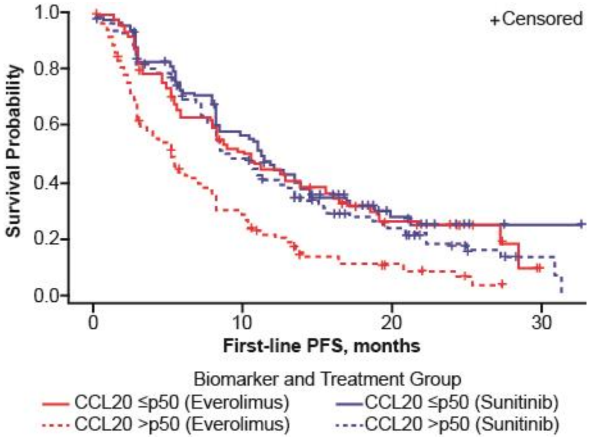

SupFIG2_08_CLEC3B

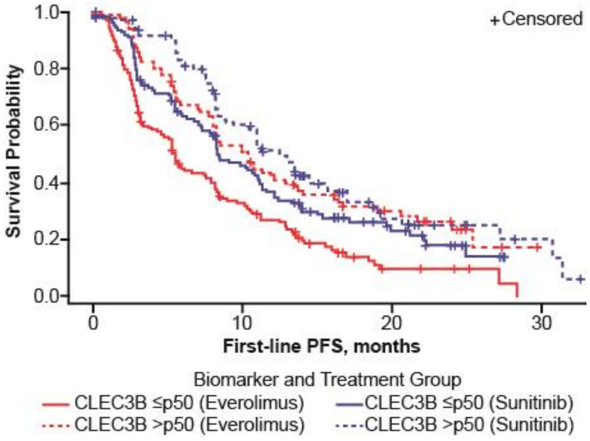

SupFIG2_09_CSF1

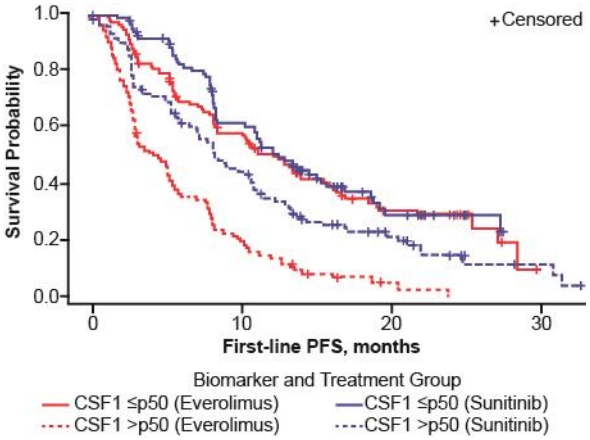

SupFIG2_10_CTSB

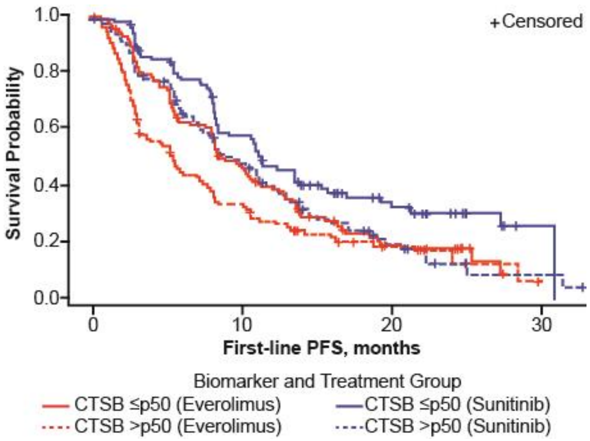

SupFIG2_11_EZR

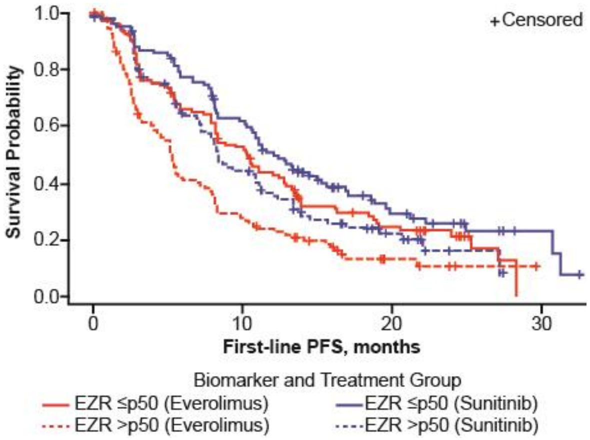

SupFIG2_12_FBLN1

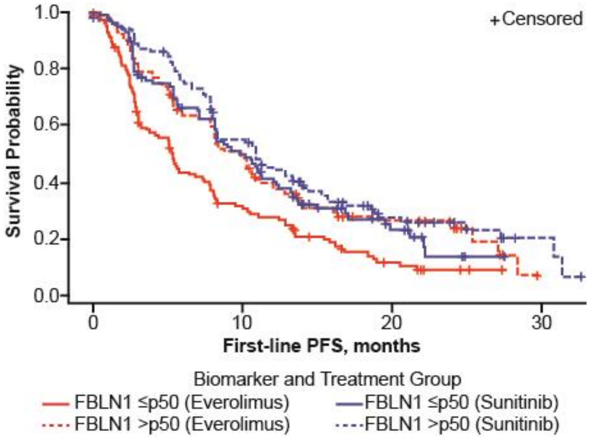

SupFIG2_13_GPI

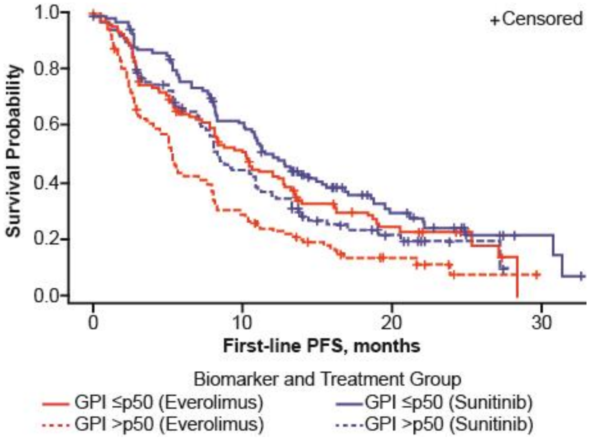

SupFIG2_14_GSN

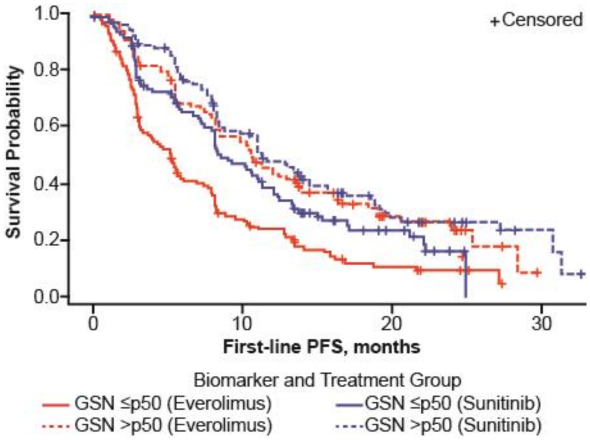

SupFIG2_15_HER2ECD

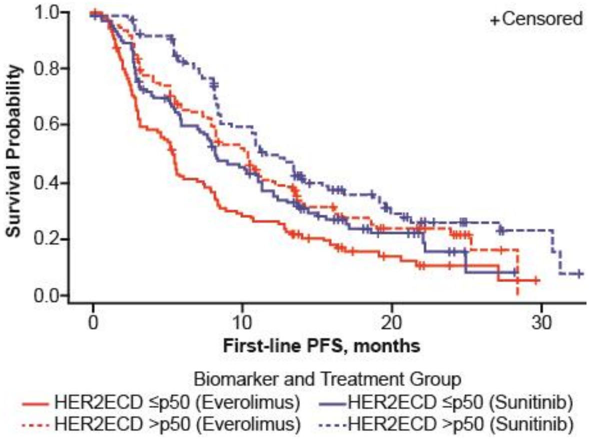

SupFIG2_16_ICAM1

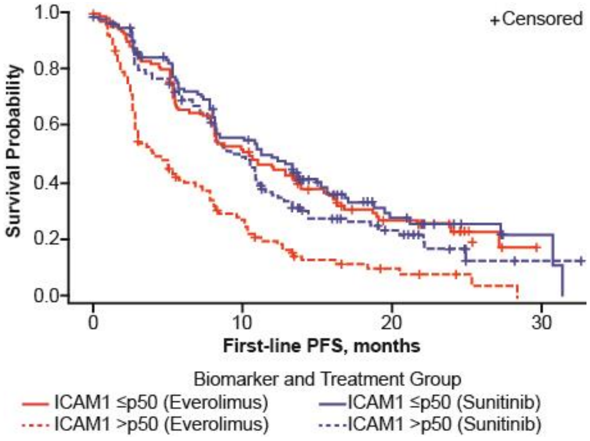

SupFIG2_17_IGFBP1

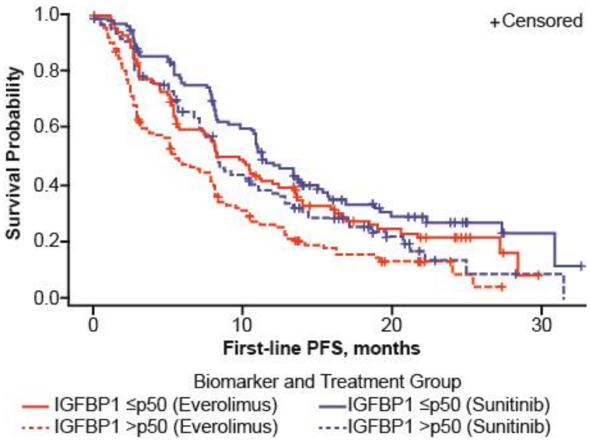

SupFIG2_18_IITCAC

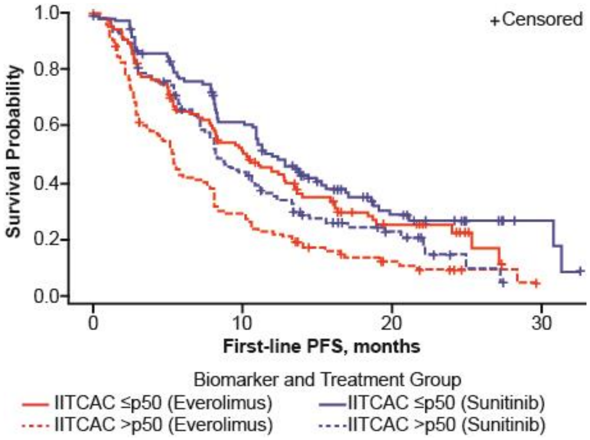

SupFIG2_19_IL10

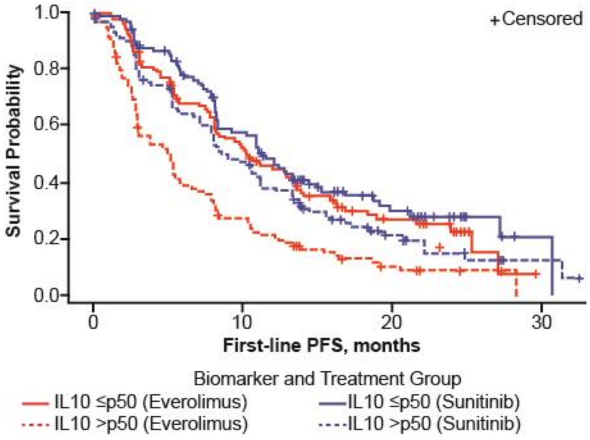

SupFIG2_20_IL18BP

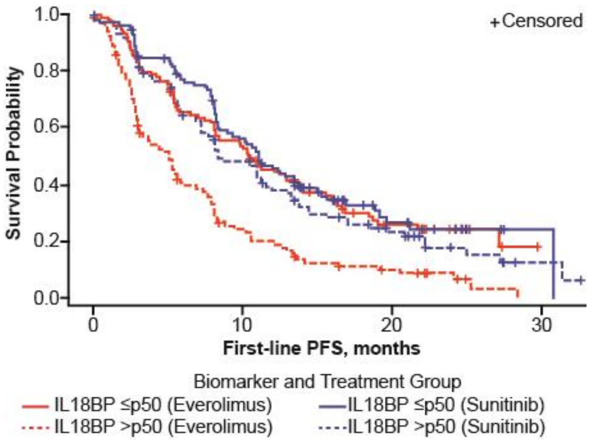

SupFIG2_21_KIM1

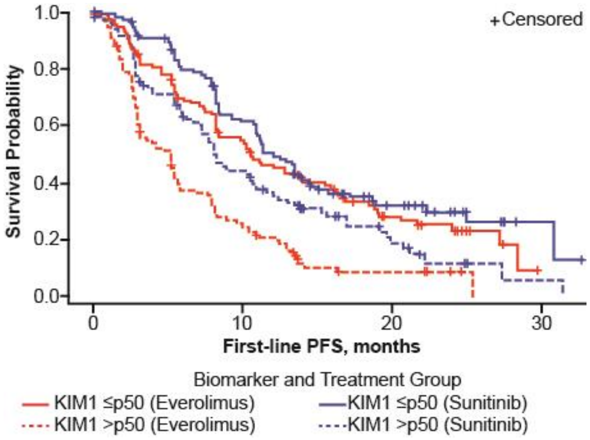

SupFIG2_22_LEPTIN

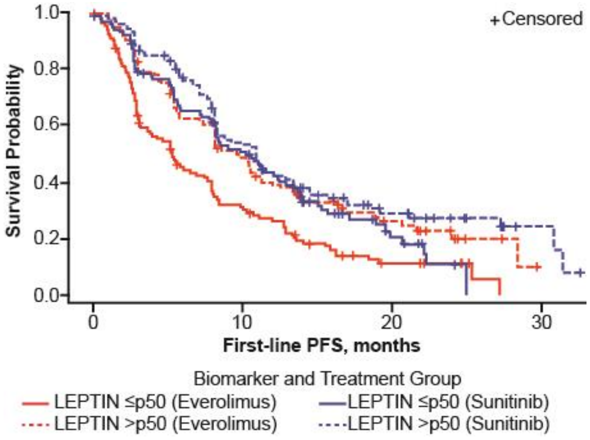

SupFIG2_23_MIP1A

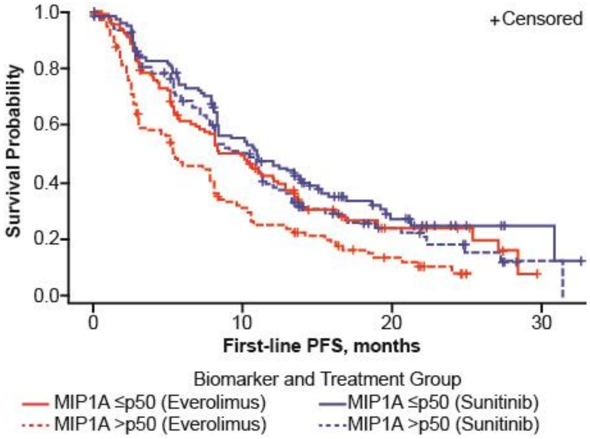

SupFIG2_24_NRP1

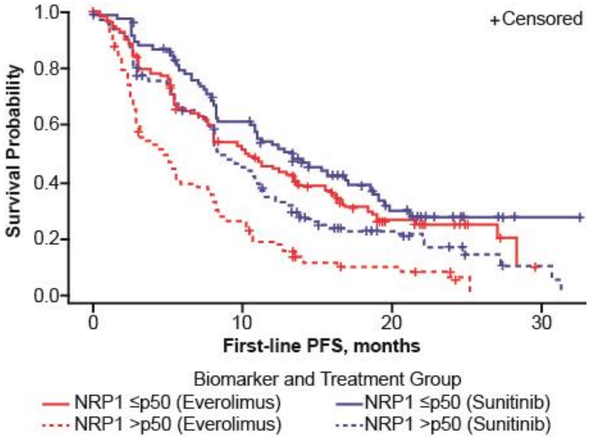

SupFIG2_25_PRL

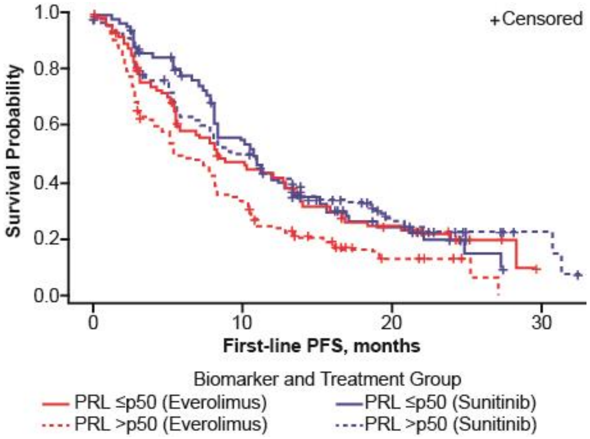

SupFIG2_26_SVEGFR3

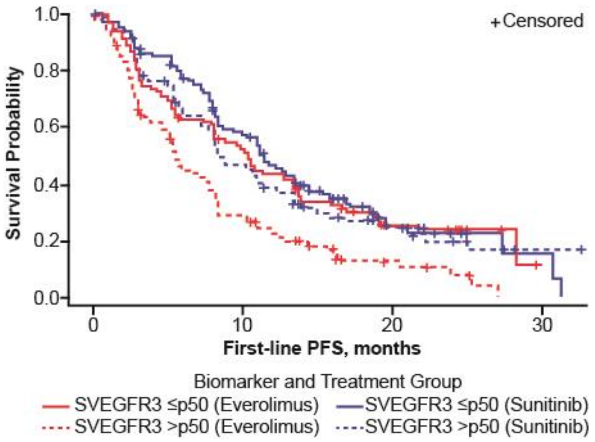

SupFIG2_27_TNFRII

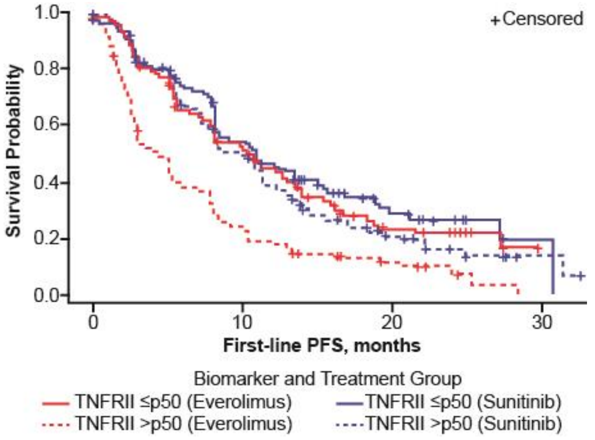

SupFIG2_28_TRAIL3

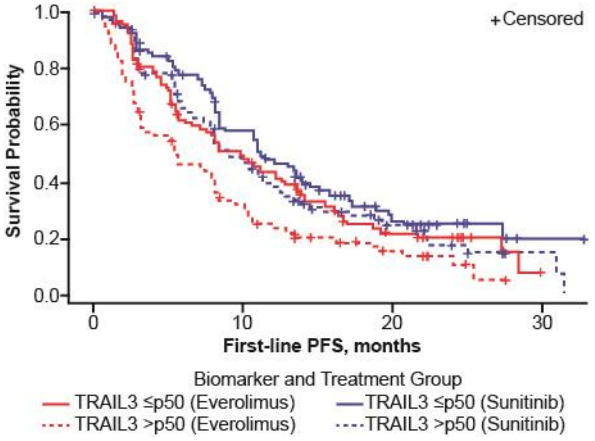

SupFIG2_29_IL18

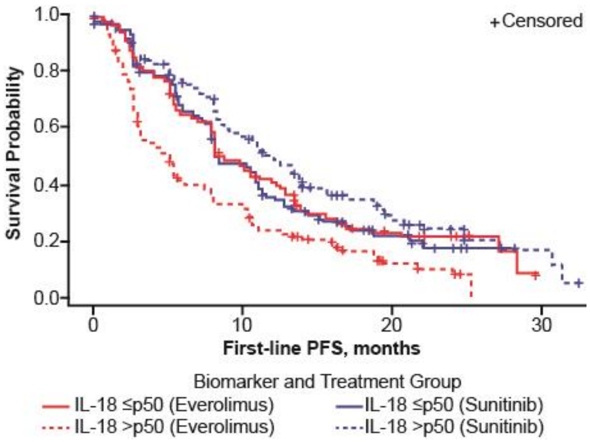

Sunitinib Predictive Biomarkers

SupFIG2_30_ANGPT1

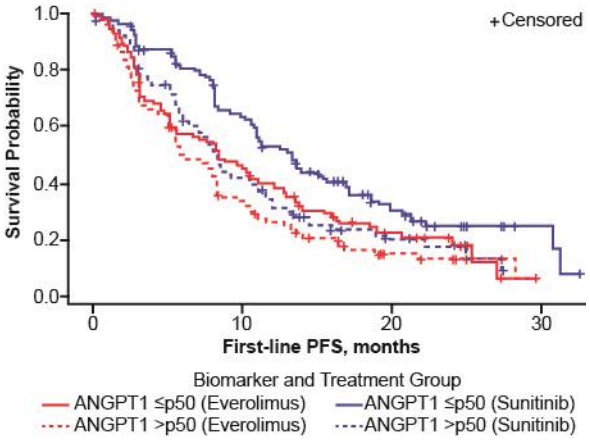

SupFIG2_31_CA153

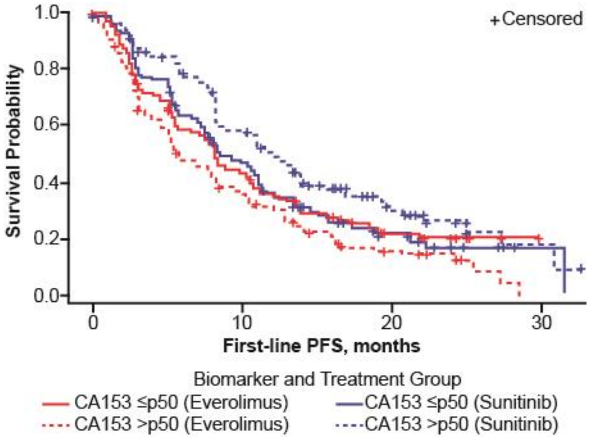

SupFIG2_32_CCL5

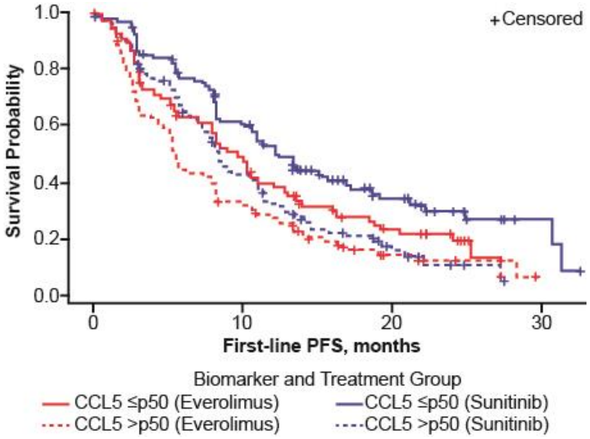

SupFIG2_33_EGFR

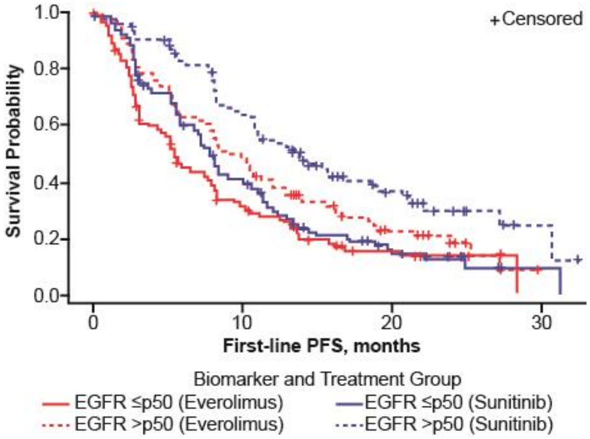

SupFIG2_34_FERRITIN

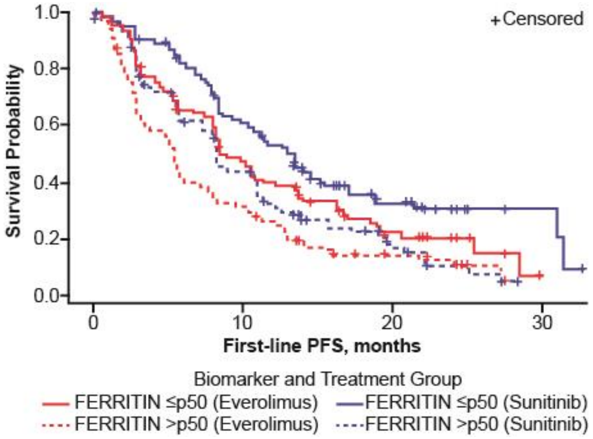

SupFIG2_35_KLK5

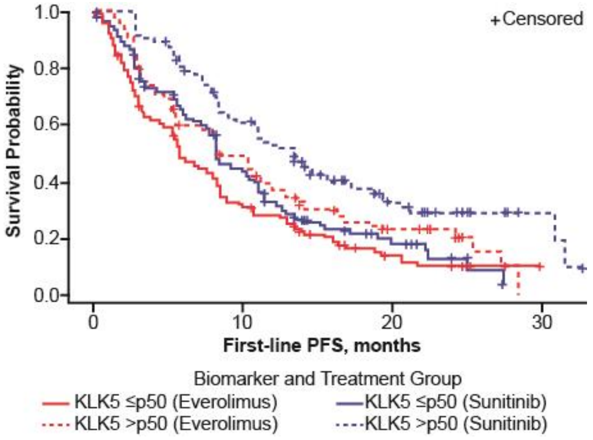

SupFIG2_36_SLPI

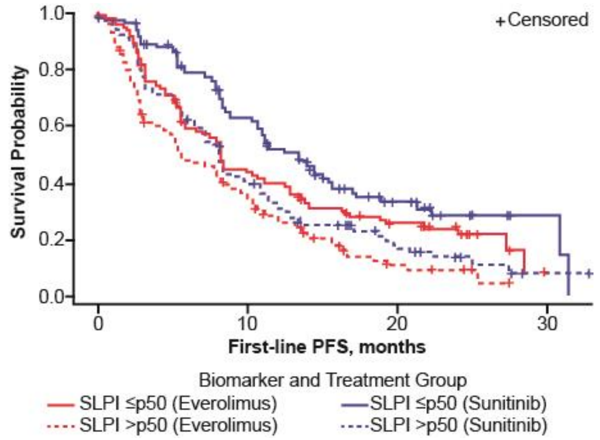

SupFIG2_37_TNC

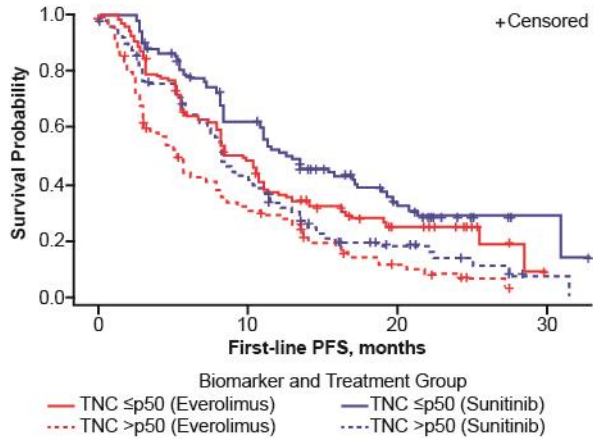

SupFIG2_38_IL18

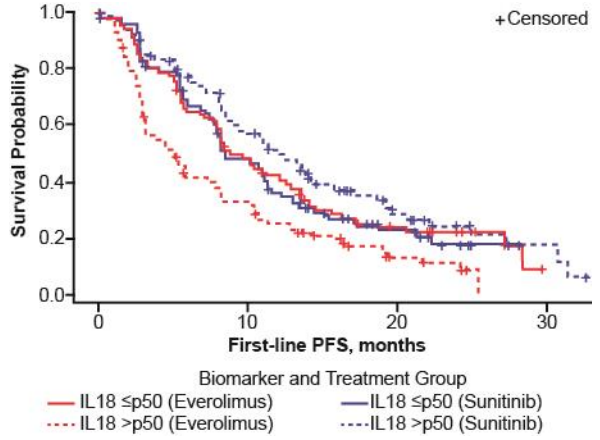

Prognostic biomarkers

SupFIG2_39_CALBIDIN

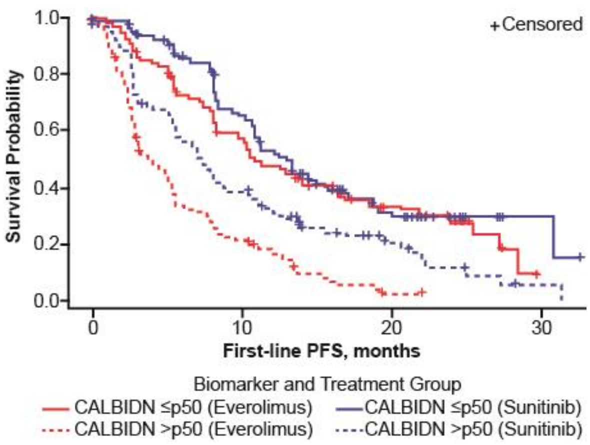

SupFIG2_40_CCL23

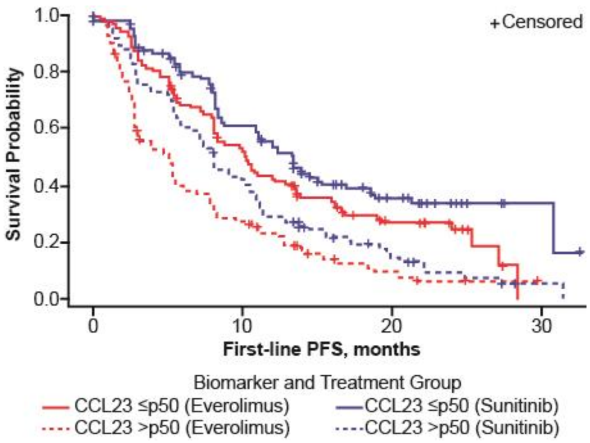

SupFIG2_41_CTSD

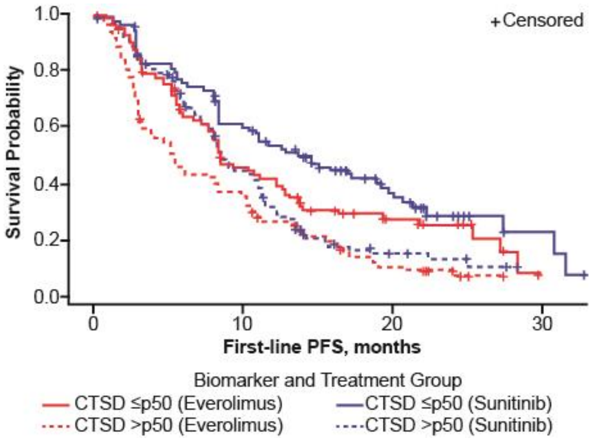

SupFIG2_42_CYSTANB

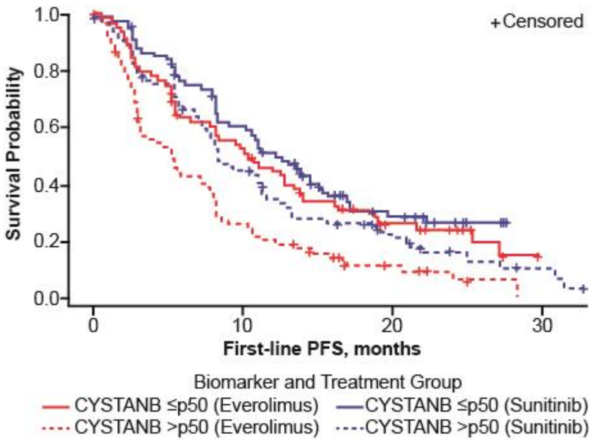

SupFIG2_43_IL-6

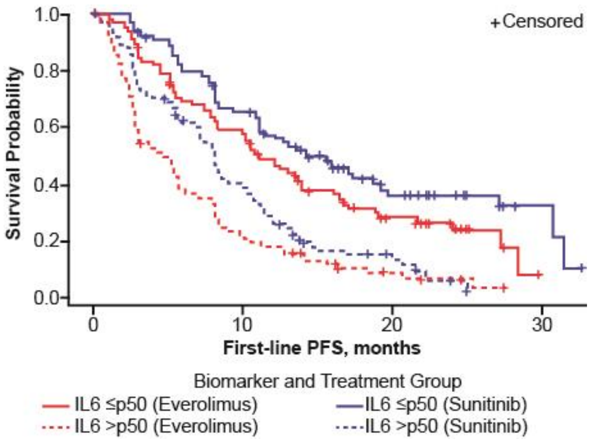

SupFIG2_44_IL8

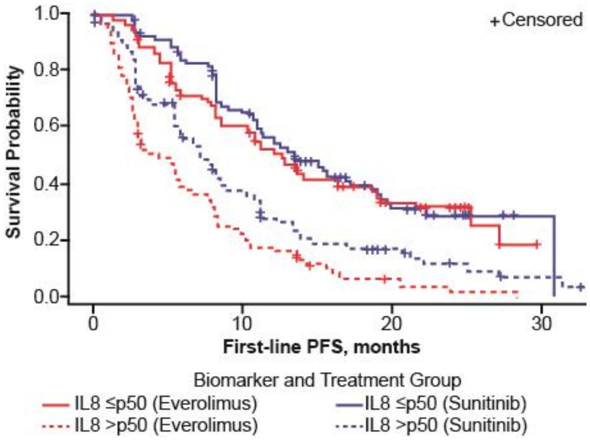

SupFIG2_45_OSTEOPTN

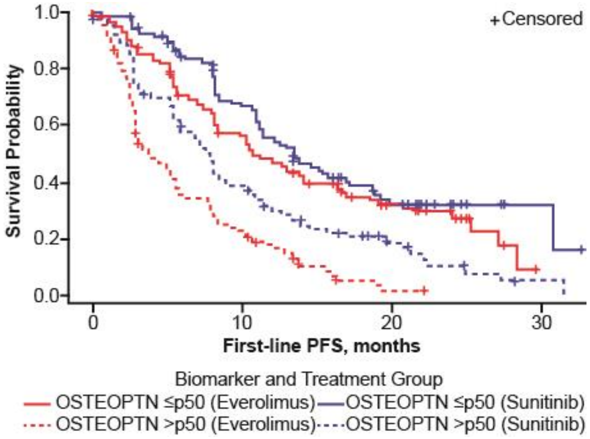

SupFIG2_46_SPINK1

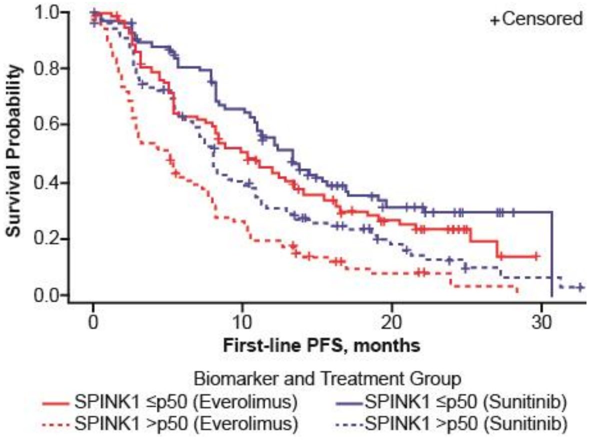

SupFIG2_47_TIMP1

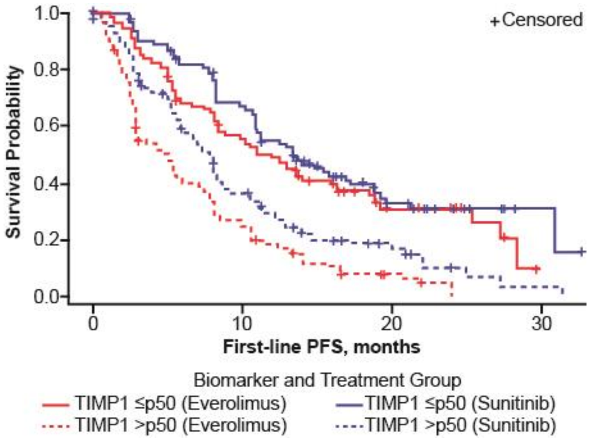

SupFIG2_48_VCAM1

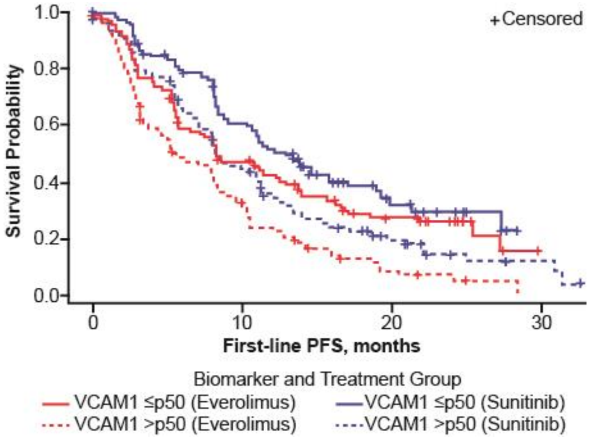

SupFIG2_49_VEGF

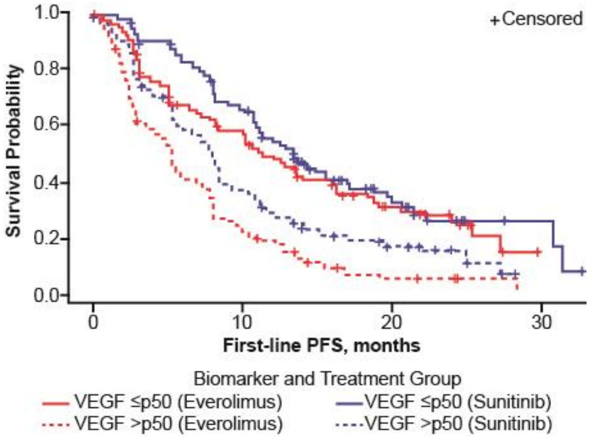

SupFIG2_50_VFDC2

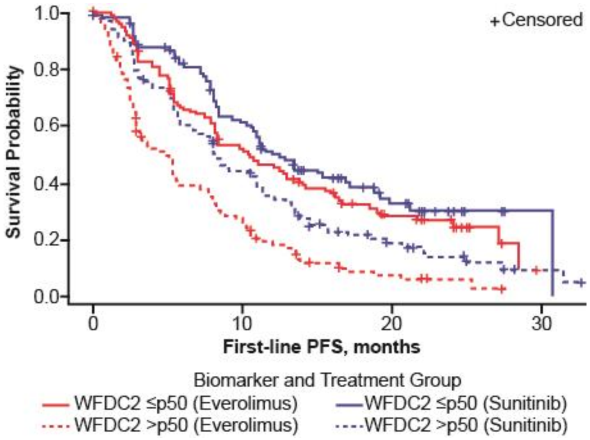

+Censored
Survival Probability
1.0
0.8
0.6
0.4
0.2
0.0
0
10
20
30
First-line PFS, months
Biomarker and Treatment Group
WFDC2 ≤p50 (Everolimus)
WFDC2 ≤p50 (Sunitinib)
WFDC2 >p50 (Everolimus)
WFDC2 >p50 (Sunitinib)
