# Supplementary material for: Circulating biomarkers and outcome from a randomised phase II trial of sunitinib vs everolimus for patients with metastatic renal cell carcinoma
Source: Br J Cancer. 2016 Feb 23;114(6):642–9. doi: 10.1038/bjc.2016.21 (PMC4800293; doi:10.1038/bjc.2016.21)
Supplement: Supplementary Information [file bjc201621x4.pdf]

SUPPLEMENTARY APPENDIX

Table 1. Biomarkers tested and included in the analyses

**Cytokine**		**Included in analyes**
Full name	*Abbreviation*	
6Ckine, CCL21	6CKINE	Yes
Alpha-2 macroglobulin	A2MGLOB	Yes
Angiotensin-converting enzyme	ACE	Yes
Adiponectin	ADPONT	Yes
Angiogenin	ANG	Yes
Angiopoietin-1	ANGPT1	Yes
Amphiregulin	AREG	No
AXL	AXL	Yes
Beta-2 microglobulin	B2MGLOB	Yes
B cell-activating factor	BCAF	Yes
Brain derived neurotropic factor	BDNF	Yes
Cancer Antigen 15-3	CA153	Yes
Cancer Antigen 72-4	CA724	Yes
Carbonic anhydrase 9	CA9	Yes
Calbindin	CALBIDN	Yes
Carcinoembryonic antigen-related cell adhesion molecule 1	CARCIEA1	Yes
Carcinoembryonic antigen-related cell adhesion molecule 6	CARCIEA6	Yes
Chemokine (C-C motif) ligand 16	CCL16	Yes
Pulmonary and activation-regulated chemokine	CCL18	Yes
Macrophage inflammatory protein 3 beta	CCL19	Yes
Macrophage inflammatory protein-3 alpha	CCL20	Yes
Myeloid progenitor inhibitory factor 1	CCL23	Yes
Chemokine (C-C motif) ligand 4	CCL4	Yes
T-cell-specific protein RANTES	CCL5	Yes
CDH1	CDH1	Yes
Cadherin 13	CDH13	Yes
YKL-40	CHI3L1	Yes
Tetranectin	CLEC3B	Yes
Collagen type IV	COL4	Yes
Macrophage colony-stimulating factor 1	CSF1	Yes
Cathepsin B (pro)	CTSB	Yes
Cathepsin D	CTSD	Yes
Stromal cell-derived factor-1	CXCL12	Yes
Cystatin-A	CYSTANA	Yes

Cystatin-B	CYSTANB	Yes
Decorin	DCN	Yes
Epidermal growth factor	EGF	Yes
Epidermal growth factor receptor	EGFR	Yes
Endoglin	ENG	Yes
Eotaxin	EOTAXIN	Yes
Epiregulin	EREG	No
Ezrin	EZR	Yes
Fatty acid-binding protein, liver	FABP1	Yes
Factor VII	FACTOR7	Yes
FASLG receptor	FASLGR	Yes
Fibulin-1C	FBLN1	Yes
Ferritin	FERRITIN	Yes
Fibroblast growth factor—basic	FGF	No
Follicle-stimulating hormone	FSH	Yes
Growth hormone	GH	Yes
Lactoylglutathione lyase	GLO1	Yes
Glucagon-like peptide 1, active	GLP1A	No
Glucagon-like peptide 1, total	GLP1T	Yes
Granulocyte/macrophage-colony stimulating factor	GMCSF	No
Glucose-6-phosphate Isomerase	GPI	Yes
Gelsolin	GSN	Yes
Heparin-binding EGF-like growth factor	HBEGF	Yes
Human epidermal growth factor receptor-2 extracellular domain	HER2ECD	Yes
Hepatocyte growth factor	HGF	Yes
Hepsin	HPN	Yes
Intercellular cell adhesion molecule	ICAM1	Yes
Interferon gamma	IFG	No
Interferon gamma induced protein 10	IFGIP10	Yes
Insulinlike growth factor-binding protein 1	IGFBP1	Yes
Interferon-inducible T-cell alpha chemoattractant	IITCAC	Yes
Interleukin-10	IL10	Yes
Interleukin-12P40	IL12P40	Yes
Interleukin-12P70	IL12P70	No
Interleukin-15	IL15	Yes
Interleukin-17	IL17	No
Interleukin-18	IL18	Yes
Interleukin-18-binding protein	IL18BP	Yes
Interleukin-1A	IL1A	No
Interleukin--1 beta	IL1B	No
Interleukin-1RA	IL1RA	Yes
Interleukin-2	IL2	No

Interleukin-23	IL23	Yes
Interleukin-3	IL3	No
Interleukin-4	IL4	No
Interleukin-5	IL5	No
Interleukin-6	IL6	No
Interleukin-6 receptor	IL6R	Yes
IL6ST	IL6ST	Yes
Interleukin-7	IL7	No
Interleukin-8	IL8	Yes
Insulin	INSULIN	Yes
Kidney injury molecule 1 (KIM-1)	KIM1	Yes
KIT	KIT	Yes
Kallikrein-5	KLK5	Yes
Kallikrein-7	KLK7	No
Lipocalin-1	LCN1	Yes
Leptin	LEPTIN	Yes
Luteinizing hormone	LHB	Yes
LTF	LTF	Yes
Monocyte chemoattractant protein 1	MCP1	Yes
Monocyte chemotactic protein 2	MCP2	Yes
Monocyte chemotactic protein 4	MCP4	Yes
Midkine	MDK	Yes
Myoglobin	MGLOBIN	Yes
Monokine induced by gamma-interferon (CXCL9)	MIG	Yes
MIP-1alpha	MIP1A	Yes
Matrix metalloproteinase 3	MMP3	Yes
Matrix metalloproteinase 9	MMP9	Yes
Mesothelin	MSLN	Yes
Beta-microseminoprotein	MSMB	Yes
Macrophage-stimulating protein	MSP	Yes
Neuropilin-1	NRP1	Yes
Osteopontin	OSTEOPTN	Yes
Plasminogen activator inhibitor 1	PAI1	Yes
PDGFB	PDGFB	Yes
Platelet endothelial cell adhesion molecule	PECAM1	Yes
Pepsinogen I	PEPSNGN1	Yes
Urokinase-type plasminogen activator receptor	PLAUR	Yes
Placenta growth factor	PLGF	Yes
Pancreatic polypeptide	PPY	Yes
Serum prolactin	PRL	Yes
PRSS8	PRSS8	Yes
Prostate-specific antigen, total	PSAT	Yes
Resistin	RETN	Yes

Protein S100-A4	S100A4	No
Soluble MET proto-oncogene (HGF receptor)	SCMET	Yes
E-Selectin	SELE	No
Maspin	SERPINB5	No
Pulmonary surfactant-associated protein D	SFTPD	Yes
Antileukoproteinase	SLPI	Yes
Pancreatic secretory trypsin inhibitor	SPINK1	Yes
Stem Cell Factor	STEMCF	Yes
Soluble vascular endothelial growth factor receptor 1	SVEGFR1	No
Soluble vascular endothelial growth factor receptor 2	SVEGFR2	Yes
Soluble vascular endothelial growth factor receptor 3 (also FLT4)	SVEGFR3	Yes
Transforming growth factor alpha	TGFA	No
Tamm-horsfall protein (THP)	THP	Yes
Tyrosine kinase with Ig and EGF homology domains 1	TIE1	No
Tissue inhibitor of metalloproteinase 1 (TIMP-1)	TIMP1	Yes
Tissue Inhibitor of metalloproteinases 2	TIMP2	Yes
Tenascin-C	TNC	Yes
Tumor necrosis factor alpha	TNFA	No
Tumor necrosis factor beta	TNFB	No
Tumor necrosis factor RII	TNFRII	Yes
Tenascin-X	TNX	Yes
TNF-related apoptosis-inducing ligand receptor 3	TRAIL3	Yes
Thyroid stimulating hormone	TSH	Yes
Vascular cell adhesion molecule 1	VCAM1	Yes
VEGF [vascular endothelial growth factor]	VEGF	Yes
Vascular endothelial growth factor B	VEGFB	No
Vascular endothelial growth factor C	VEGFC	Yes
Vascular endothelial growth factor D	VEGFD	No
HE4	WFDC2	Yes

Table 2. Multivariate analysis first-line PFS, including CBS by treatment arm

**Effect**	***P***
Treatment (everolimus)	<0.0001
CBS (>3)	0.1399
CBS by treatment	0.0321
Histology (clear cell)	0.3159
Metastatic sites (>1)	<0.0001
Baseline LDH value	0.0875

Abbreviation: CBS, composite biomarker score.

CBS is dichotomized as low (≤ 3) and high (>3) using median CBS score.

Table 3. Interaction between MSKCC risk category and CBS

MSKCC	Treatment	CBS	No. of patients and events	Median PFS 1L, months	95% CI for median	
Favorable	EVE	High	44/26	15.87	11.07	27.17
	EVE	Low	36/27	8.28	5.26	11.99
	SUN	High	30/17	15.67	11.01	30.82
	SUN	Low	49/31	13.37	10.12	19.19
Intermediate or Poor	EVE	High	41/28	10.71	5.62	16.59
	EVE	Low	105/93	3.58	2.89	5.36
	SUN	High	36/21	12.02	NE	NE
	SUN	Low	101/81	8.08	5.85	9.00

MSKCC	Comparison of Biomarker	Within	Cox PH model			Log-rank test	
			HR	95% CI for HR		Raw *P* value	FDR *P* value
Favorable	EVE vs SUN	High	1.033	0.557	1.918	0.4879	0.4879
	EVE vs SUN	Low	2.142	1.250	3.669	0.0193	0.0385
Intermediate or Poor	EVE vs SUN	High	1.356	0.757	2.429	0.2689	0.2689
	EVE vs SUN	Low	2.072	1.523	2.819	0.0001	0.0002
Favorable	High vs low	EVE	0.429	0.246	0.747	0.0047	0.0094
	High vs low	SUN	0.889	0.484	1.633	0.3492	0.3492
Intermediate or Poor	High vs low	EVE	0.399	0.260	0.611	<0.0001	<0.0001
	High vs low	SUN	0.609	0.368	1.008	0.0099	0.0099

Abbreviatios: CBS, composite biomarker score; MSKCC, Memorial Sloan Kettering Cancer Center; NE, not evaluable.

CBSis dichotomized as low (≤ 3) and high (>3) using median. CBS score *P* value is adjusted only at the CBS level, across multiple cutoffs and multiple comparisons via the Benjamini–Hochberg FDR adjustment.
